# AI-driven pilot platforms and computational pharmaceutics: accelerating innovation in small molecule drug development under industry 4.0 and 5.0 paradigms

**DOI:** 10.3389/fphar.2026.1681040

**Published:** 2026-03-04

**Authors:** Kaixin Luo, Yuhan Yang, Sadaruddin Chachar, Chenggong Zhong, Meiqi Chen, Jun Xiong, Lianyi He, Dingying Liu, Shahla Karim Baloch, Ihab Elshoura, Zaid Chachar, Yuanzhe Cai, Feijuan Huang

**Affiliations:** 1 Shenzhen Institute of Translational Medicine, The First Affiliated Hospital of Shenzhen University, Shenzhen Second People’s Hospital, Shenzhen, China; 2 Shenzhen Technology University, Shenzhen, China; 3 Shenzhen Weimei Medical Devices Co. Ltd., Shenzhen, China; 4 Department of Biotechnology, Sindh Agriculture University, Tandojam, Pakistan; 5 Nanjing University, Nanjing, China; 6 The Chinese University of Hong Kong Shenzhen, Shenzhen, China

**Keywords:** artificial intelligence, industry 4.0, pilot-scale platform, small molecule drugs, smartpharmaceutical manufacturing

## Abstract

In the era of artificial intelligence (AI) and Industry 4.0, pilot-scale platforms for small molecule chemical drugs are undergoing a transformative digital evolution. These platforms serve as a critical link between early-stage laboratory research and full-scale pharmaceutical manufacturing, ensuring process feasibility, scalability, and regulatory compliance. This review offers a comprehensive and forward-looking analysis of the structure, function, and strategic importance of pilot-scale systems within the modern pharmaceutical landscape. Focusing on the integration of AI and intelligent automation, the study highlights innovations such as AI-driven process optimization, predictive maintenance, data integration, digital twin technologies, and continuous manufacturing. These technologies are reshaping conventional production paradigms by enhancing efficiency, improving quality control, and reducing environmental impact. The convergence of computational pharmaceutics and green chemistry is also examined as a key driver of sustainable and intelligent drug development. Moreover, the review addresses the industry’s transition toward Industry 5.0, characterized by human-machine collaboration, data-centric innovation, and an emphasis on sustainability. Persistent challenges such as equipment standardization gaps, data-sharing limitations, and outdated infrastructure are critically discussed. Drawing from industrial case studies, academic research, and best practices, this paper explores both the opportunities and constraints associated with AI-enabled pilot platforms. Ultimately, the review aims to inform future strategies in digital pharmaceutical manufacturing by underscoring the importance of technological innovation, regulatory alignment, and collaborative ecosystems in advancing the development, efficiency, and sustainability of small molecule drug production.

## 1 Introduction

Small molecule chemical drugs have long constituted the cornerstone of modern pharmacotherapy, owing to their well-defined physicochemical properties and the maturity of their chemical production systems (Segler and Waller, 2017). Despite their clinical significance, the transition of candidate drug molecules from laboratory synthesis to industrial-scale production presents substantial challenges. This critical stage in the pharmaceutical development pipeline, particularly at the pilot-scale level entails the translation of research outputs into scalable, stable, and regulatory-compliant processes. However, this phase is often fraught with high levels of risk, prolonged timelines, and significant financial burdens (Schwaller et al., 2019). Traditional pilot-scale operations have historically relied on empirical methodologies and manual expertise. Such reliance limits predictive capability and process adaptability, making it difficult to ensure reproducibility and efficiency when scaling up. Key issues commonly encountered include poor scalability forecasting, underutilization of valuable process data, and excessive consumption of time and resources. Amidst these challenges, the convergence of artificial intelligence (AI) with Industry 4.0 technologies is revolutionizing the design and execution of pilot-scale pharmaceutical platforms. These intelligent systems offer novel solutions for enhancing process robustness, streamlining workflows, and enabling more precise decision-making through advanced data analytics. The incorporation of AI-driven strategies into pilot-scale development aims not only to optimize scalability and reduce operational risk but also to expedite development timelines and improve access to novel therapeutics (Han et al., 2022a). By leveraging these digital tools, pharmaceutical manufacturing is poised to enter a new era of innovation, characterized by automation, efficiency, and adaptability.

This review followed a simplified PRISMA-style approach to ensure transparent and systematic coverage of the literature. Searches were conducted between January and March 2025 using PubMed, Scopus, Web of Science, and Google Scholar. The search terms included combinations of “pilot-scale pharmaceutical manufacturing,” “AI in drug development,” “Industry 4.0,” “computational pharmaceutics,” “digital twins,” “continuous manufacturing,” and “automation.”

The initial search identified 1,284 records. After removing duplicates, 943 articles remained. Titles and abstracts were screened to exclude papers unrelated to small molecule drug development, pilot-scale processes, or digital manufacturing technologies. Full-text screening was applied to 267 articles, and 158 met the inclusion criteria. Additional relevant sources, including industrial white papers and regulatory guidance documents (FDA, EMA, ICH), were included through manual cross-referencing to ensure comprehensive coverage of technological developments. Only peer-reviewed studies, high-quality reviews, and authoritative industrial or regulatory publications were included. Studies focused solely on biologics manufacturing, exclusively clinical outcomes, or unrelated computational modeling were excluded. This method ensured that the evidence synthesized in this review reflects the most relevant and up-to-date advancements at the intersection of AI, pilot-scale platforms, and modern pharmaceutical manufacturing.

To move beyond conceptual discussion, this review integrates concrete pilot-scale case examples, named AI tools, and implementation-level methodologies drawn from validated academic and industrial reports. These include AI-assisted synthesis planning systems, pilot-scale continuous manufacturing case studies, real-time process analytical technology deployments, and human-in-the-loop control architectures, providing practical context for how AI is applied in contemporary pharmaceutical development. This review also addresses a single central question: How can artificial intelligence and digital technologies strengthen pilot-scale platforms to accelerate and de-risk small molecule drug development? To answer this, the review synthesizes evidence across three tightly connected themes: (1) the role of AI-enabled computational pharmaceutics in guiding formulation and synthesis decisions, (2) the contribution of smart and continuous manufacturing technologies to pilot-scale reliability and quality, and (3) the integration of digital twins and predictive analytics to improve scalability and operational robustness. By consolidating these strands into one focused framework, the review clarifies how emerging digital tools collectively transform the pilot stage into a more efficient, data-driven, and sustainable engine for small molecule drug development. By integrating insights from computational pharmaceutics, green chemistry, and smart manufacturing, the review seeks to guide the strategic transformation of pilot-scale systems toward more intelligent, efficient, and environmentally responsible pharmaceutical production.

### 1.1 Current status of small molecule chemical drug development

Small molecule drugs continue to play an indispensable role in clinical therapeutics due to their distinctive pharmacological characteristics. Their ability to efficiently permeate cell membranes and directly interact with intracellular targets enables precise drug delivery to affected tissues and cells (Southey and Brunavs, 2023), (Beck et al., 2022). Compared to biologic therapies, small molecule drugs generally exhibit more predictable pharmacokinetic and pharmacodynamic profiles, greater chemical stability, and lower immunogenicity. Moreover, their oral bioavailability and ease of administration enhance patient compliance and broaden their clinical applicability (Prueksaritanont and Tang, 2012). These advantages have made small molecule drugs a mainstay in the treatment of a wide range of diseases, from cancer to autoimmune and infectious disorders. For example, Vitrakvi, approved by the U.S. Food and Drug Administration (FDA) in 2018, was among the first tumor-agnostic therapies, indicated for various solid tumors with NTRK gene fusions. Similarly, umbralisib has demonstrated significant therapeutic efficacy in patients with recurrent or refractory marginal zone lymphoma (MZL), follicular lymphoma (FL), and small lymphocytic lymphoma (SLL), attributed to its dual inhibitory action on phosphatidylinositol 3-kinase δ (PI3Kδ) and casein kinase 1ε (CK1ε) (Gu et al., 2023).

**FIGURE 1:**
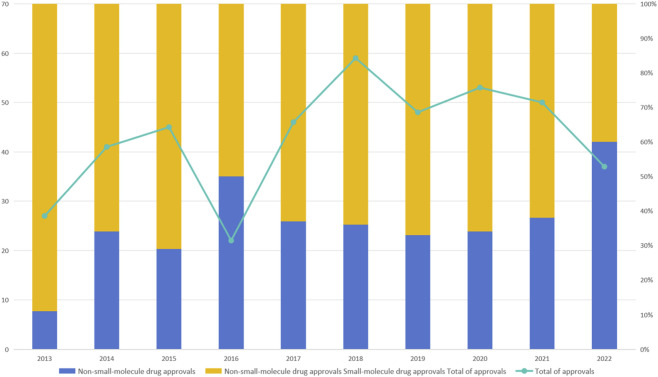
Annual drug approvals by the U.S. Food and Drug Administration (FDA) from 2013 to 2022, categorized by drug type. The bar segments represent the number of small molecule drug approvals (yellow) and non-small-molecule drug approvals (blue) each year. The line graph (green) indicates the total number of annual drug approvals. The figure highlights the consistently dominant contribution of small molecule drugs to the total approvals over the past decade, emphasizing their continued importance in pharmaceutical innovation. Data source: U.S. Food and Drug Administration (https://www.fda.gov/drugs/development-approval-process-drugs/novel-drug-approvals-fda).

These examples underscore the irreplaceable role of small molecule drugs in contemporary medicine and highlight their considerable potential in both personalized and targeted therapeutic strategies. Data over the past decade consistently demonstrate that small molecule chemical drugs constitute a dominant share of newly approved pharmaceutical products each year, underscoring their ongoing relevance across diverse disease areas and clinical applications (Figure 1) (https://www.fda.gov/drugs/development-approval-process-drugs/novel-drug-approvals-fda). The figure demonstrates that from 2013 to 2022, small molecule drugs have accounted for more than half of all annual approvals by the U.S. Food and Drug Administration (FDA), with certain years such as 2018 and 2022 exhibiting particularly high proportions. This trend confirms the sustained innovation and adaptability of small molecule platforms within evolving drug development pipelines. Furthermore, as researchers gain deeper insights into molecular structures and interaction mechanisms, there is a growing opportunity to revisit and repurpose drug candidates that were previously discontinued during clinical trials. Through structural modification and functional optimization, these precursor molecules can be redesigned to enhance efficacy, reduce toxicity, or target alternative disease pathways. Such strategies not only breathe new life into abandoned compounds but also maximize the return on earlier R&D investments. This lifecycle extension approach contributes to a more efficient and sustainable model of pharmaceutical innovation.

**FIGURE 2:**
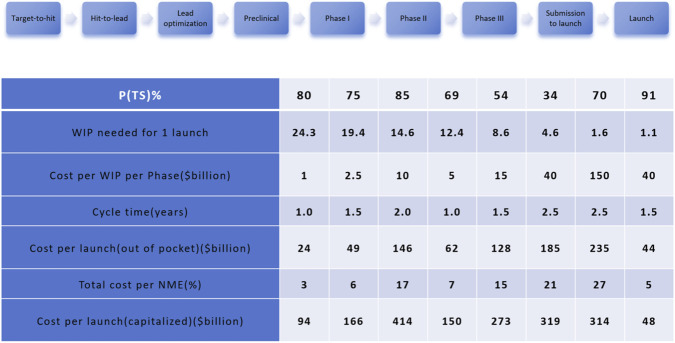
Economic model of the drug development process for a new molecular entity (NME). The figure outlines key stages from target identification to product launch, including transition probabilities [P (TS)%], work-in-process (WIP) required for one launch, cost per WIP per phase (in billions USD), average cycle time (in years), and both out-of-pocket and capitalized costs per launch. Later-stage clinical trials (Phases II and III) contribute disproportionately to total development cost and timeline. Adapted from Paul et al. (2010).

However, the economic burden associated with bringing a small molecule drug from laboratory discovery to commercial launch remains substantial, with recent analyses estimating average capitalized R&D costs of roughly USD 2–3 billion per approved new molecular entity when the costs of failures and time value of money are included (Segler and Waller, 2017), (DiMasi et al., 2016). This high expenditure is largely driven by two primary factors: first, the exceptionally low success rate of drug candidates during clinical development, where more than 85% ultimately fail, which significantly inflates the overall development cost (Wong et al., 2019); and second, the inherent complexity and duration of the drug discovery and development process, which requires extensive human, technological, and financial resources over prolonged periods. To mitigate these challenges, strengthening the pipeline of viable preclinical candidates is vital for enhancing downstream success rates and reducing attrition across later development phases. The comprehensive economic model proposed by Paul et al. (2010) offers a detailed visualization of the costs, timelines, and probabilities associated with each phase of the new molecular entity (NME) development process (Figure 2). The figure delineates nine sequential stages ranging from target identification to final launch and quantifies key metrics such as transition probabilities [P (TS)%], work-in-process (WIP) requirements, cost per WIP per phase, average cycle time, and both out-of-pocket and capitalized costs per launch. As the model illustrates, later-stage clinical trials (particularly Phases II and III) are associated with high per-phase expenditures and prolonged timelines, despite relatively lower probabilities of success. For example, Phase III alone accounts for a capitalized cost on the order of several hundred million U.S. dollars per successful launch, reflecting both its financial intensity and its impact on overall R&D efficiency. The submission-to-launch and launch phases also demonstrate high capitalized costs due to regulatory and commercialization complexities. Consequently, optimizing early-stage candidate selection and improving decision-making frameworks throughout the pipeline are essential to balancing cost, time, and success probability an enduring challenge in contemporary pharmaceutical R&D.

### 1.2 Pilot platforms in the drug development process

**FIGURE 3:**
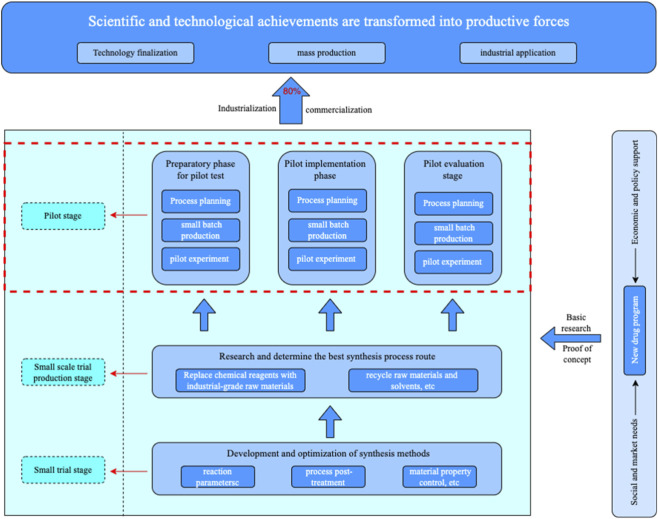
Flowchart of the drug development process from small trial stage to industrial application. The figure outlines the structured transition from laboratory-scale experimentation to pilot-scale validation and eventually to mass production. Key phases include the small trial stage, small-scale trial production, and the pilot stage subdivided into preparatory, implementation, and evaluation phases. Each sub-phase involves process planning, small-batch production, and pilot testing. The framework highlights the iterative refinement of synthesis processes, substitution of lab-grade with industrial-grade materials, and emphasizes the role of economic, policy, and societal support in advancing new drug programs. The transformation success rate for technologies validated through pilot platforms is noted to reach up to 80%.

The pilot phase represents a pivotal stage in the pharmaceutical development lifecycle, acting as a critical bridge between laboratory research and full-scale industrial production (Figure 3) (Zhang and Zhang, 2023). While the small-scale phase is primarily dedicated to method development and optimization under tightly controlled laboratory conditions, the pilot-scale phase aims to validate these methods using equipment and processes that closely resemble actual manufacturing environments. This validation ensures that the developed processes are not only reproducible but also robust enough to consistently meet quality standards under industrial-scale conditions (Vasconcellos et al., 2021).

The key distinctions between laboratory and pilot scales lie in material quantities, equipment configurations, and operational parameters. Laboratory-scale experiments typically involve small batches and emphasize fine-tuning reaction conditions. In contrast, pilot-scale operations handle larger volumes of materials and rely on production-representative systems to simulate industrial processes. At this stage, critical engineering factors such as heat transfer efficiency, mixing dynamics, and reactor construction materials are closely examined to assess the scalability and industrial applicability of the process (Zhang and Zhang, 2023), (Lab-Scale Experiment, 2025).

Pilot testing has been shown to significantly enhance the success rate of technology transfer, with transformation success rates reaching up to 80% for processes that undergo pilot validation, compared to just 30% for those that do not (Shandong Institute of I ndustrial Technology for Health Sciences and Medicine, 2025). Beyond technical validation, pilot platforms serve a broader strategic role by facilitating the commercialization of emerging technologies and fostering collaborations between academic institutions and industry stakeholders. These platforms also play a crucial role in aligning technological innovation with market demand, supporting the formation of high-quality industrial clusters, optimizing enterprise infrastructure, and accelerating regional innovation ecosystems (Zhao et al., 2023). In this context, pilot platforms emerge as essential instruments for advancing industrial innovation and integrating scientific research with economic development.

The structural framework and strategic progression of the pilot phase are further explained in Figure 3, which delineates the stages through which scientific and technological achievements are transformed into viable industrial applications. The pilot stage is composed of three main sub-phases: the preparatory phase, the implementation phase, and the evaluation phase. Each of these includes process planning, small-batch production, and pilot experimentation designed to simulate and optimize real-world production conditions. The figure also emphasizes the transition from the small trial and small-scale trial production stages to the pilot stage, where chemical reagents are replaced with industrial-grade raw materials, and the recycling of solvents and raw materials is initiated. The development begins with the optimization of reaction parameters, post-treatment processes, and material property control, laying the groundwork for subsequent scale-up. The goal of the pilot platform is to refine the synthesis process to such a degree that mass production becomes technically feasible, economically viable, and regulatorily compliant. Notably, processes that progress through structured pilot validation show a transformation success rate as high as 80%, reinforcing the value of this intermediate phase in bridging innovation and application.

Furthermore, the figure reflects the interplay between economic, policy, and market factors that drive and support pilot activities. New drug programs are not only technology-driven but are also shaped by broader ecosystem influences, including regulatory frameworks and societal demand.

**FIGURE 4:**
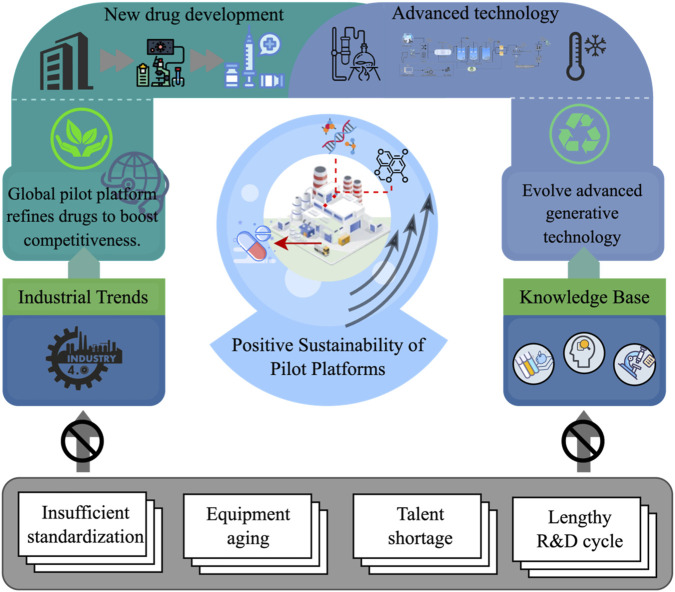
Conceptual framework exemplifies the sustainability and advancement of global pilot platforms in pharmaceutical development. The figure highlights the central role of pilot platforms in refining drug production and promoting competitiveness, supported by Industry 4.0, generative technologies, and knowledge-based innovation. Key enablers include advanced equipment and data integration, while core challenges such as insufficient standardization, equipment aging, talent shortages, and long R&D timelines are identified as limiting factors. The model emphasizes a feedback loop wherein technological and strategic improvements reinforce the sustainable evolution of pilot-scale systems.

The global emergence and advancement of pilot platforms, largely driven by the principles of Industry 4.0, have significantly enhanced both the efficiency and quality of pharmaceutical development processes. To maintain competitiveness in a rapidly evolving market, leading pharmaceutical companies and research institutions are increasingly investing in the establishment and modernization of these platforms (Tianfu International Biocity, 2025). As shown in Figure 4, cutting-edge pilot platforms are incorporating advanced technologies such as integrated continuous manufacturing (ICM), freeze-dissolving technology, and supercritical fluid extraction (SFE). These innovations have not only boosted production throughput but also contributed to substantial reductions in operational costs (Supercritical fluid, 2021; Hyer et al., 2024; Luo et al., 2024). Despite these technological advancements, many pilot platforms still face persistent challenges.

Key limitations include the lack of standardized equipment and process protocols, reliance on outdated infrastructure, and inefficiencies in management systems. These issues continue to hamper the scalability, reliability, and regulatory compliance of drug development operations at some sites, thereby constraining their full potential (Jiang and Cao, 2022), (Haigney, 2022), (Sarkis et al., 2021). As chemical manufacturing processes scale up, the integration of green chemistry principles at the pilot stage has become increasingly prominent. The widespread adoption of catalytic reactions and solvent substitution strategies has led to notable improvements in production efficiency while simultaneously reducing the environmental footprint of pharmaceutical manufacturing (Green Chemistry, 2024). For instance, Yang et al. reported a cobalt-catalyzed enantioselective hydrogenation method that enhanced reaction selectivity and minimized the formation of by-products (Yang et al., 2023). Similarly, Sun et al. developed a nickel-catalyzed transfer hydrogenation protocol that achieved higher product yields with improved selectivity (Sun et al., 2023). These innovations underscore the potential of green catalysis to elevate both the environmental and operational performance of pilot-scale systems.

In addition, biocatalysis is emerging as a powerful approach in sustainable synthesis. The work of Harwood et al. demonstrated the use of engineered P450BM3 enzymes to facilitate stereoselective synthesis of chiral compounds an essential class of molecules in pharmaceutical applications (Harwood et al., 2023). Such developments in biocatalytic technology offer a promising avenue for reducing the environmental impact of chemical processes, further solidifying the role of green chemistry in the future of drug development.

However, furthermore as demonstrated in Figure 4, the global rise of pilot platforms is being shaped by a dynamic interplay of industrial trends, technological advancements, and knowledge-driven innovation. The figure conceptualizes the role of pilot platforms as a central force in enhancing the sustainability and competitiveness of pharmaceutical development. It highlights how Industry 4.0 technologies and evolving generative technologies drive the modernization of pilot systems, while also drawing on enriched knowledge bases to support innovation. At the core of this model is the positive sustainability loop generated by pilot platforms. These platforms not only accelerate new drug development but also refine production practices, boosting industrial competitiveness on a global scale. However, the figure also acknowledges persistent systemic challenges, such as insufficient standardization, aging infrastructure, workforce shortages, and lengthy R&D cycles. These barriers can inhibit the full realization of pilot platforms’ potential and must be addressed through coordinated investment in technology, human capital, and regulatory reform. Ultimately, the figure underscores the need for a holistic strategy one that integrates cutting-edge technologies, knowledge-sharing ecosystems, and long-term policy support to maximize the value of pilot platforms and ensure their sustainability in the era of smart pharmaceutical manufacturing.

To address the persistent challenges facing pilot-scale drug development, the pharmaceutical industry is increasingly transitioning from traditional batch-based methods to continuous manufacturing paradigms (Lee et al., 2015). Among these, end-to-end integrated continuous manufacturing (ICM) has emerged as a transformative trend, driven by the dual imperatives of process efficiency and technological innovation. ICM facilitates enhanced product quality through real-time monitoring and control, leveraging online sensors and automated feedback systems to maintain process consistency. Simultaneously, it contributes to reduced operational costs and energy consumption, fostering greater standardization across manufacturing platforms (Orehek et al., 2021). In parallel, the evolution of the smart factory a foundational concept within Industry 4.0, reshaping the pharmaceutical production landscape. Smart factories integrate data analytics, advanced automation, and artificial intelligence to enable real-time process monitoring, predictive maintenance, and adaptive control. These capabilities significantly enhance production efficiency and ensure quality stability across the manufacturing cycle (Anthwal et al., 2024).

As the smart factory model evolves, pharmaceutical manufacturing is moving toward Industry 5.0, where human expertise and advanced digital systems operate together rather than replacing one another. This transition is already visible in several practical applications. Human-in-the-loop advanced process control (APC) systems are being deployed in continuous tableting and biologics fill-finish operations, where operators supervise AI-generated adjustments to critical parameters such as granulation temperature, screw speed, or coating uniformity to ensure GMP compliance. Explainable AI tools are also gaining adoption in areas such as real-time release testing (RTRT), where chemometric models Raman PCA, NIR-PLS, and hybrid neural networks—must provide interpretable outputs to satisfy regulatory requirements (Rada, 2015; Rada, 2020). In addition, cybersecurity has become a central operational concern as IIoT-enabled factories introduce risks such as sensor spoofing, data-integrity compromise, and unauthorized control-system access (Coelho et al., 2023). Recent FDA and EMA guidance now require formal cyber-risk mitigation strategies for digital manufacturing systems, including multi-layer authentication, encrypted data flows, and audit-ready traceability. These developments demonstrate that Industry 5.0 in pharma is not an abstract ideal but an applied framework where human judgement, interpretable AI, and resilient cyber-physical systems work together to strengthen quality and operational robustness (van Erp et al., 2024). In the pharmaceutical sector, Industry 5.0 facilitates applications such as digital twins for inventory management, supply chain simulation, and disruption prediction, enhancing resilience and personalization (Maddikunta et al., 2022). The shift from automation-focused models to those centered on synergistic human involvement marks a critical step in driving long-term innovation, operational resilience, and sustainable development within the pharmaceutical sector (Markey et al., 2024).

## 2 Main body

### 2.1 Current developments in computational pharmacy

Computational pharmaceutics represents a multidisciplinary field that merges principles from pharmacy, chemistry, biology, computer science, and engineering. Since the early 2010s, discipline has entered a new era, fueled by the exponential growth of big data and bioinformatics, alongside the widespread integration of machine learning (ML) and artificial intelligence (AI) technologies. These innovations have significantly advanced the capabilities and applications of computational pharmaceutics, enabling a more efficient and informed approach to drug development (Markey et al., 2024), (Sadybekov and Katritch, 2023). Through the incorporation of multi-scale simulation models, AI algorithms, and big data analytics, computational pharmaceutics has shifted the paradigm of pharmaceutical research from traditional trial-and-error experimentation to data-driven and predictive modeling (Mitra et al., 2024). This transition allows for the integration of diverse computational methods—such as molecular modeling, optimization algorithms, and predictive simulations—into formulation development processes (Wang et al., 2021a), (Suriyaamporn et al., 2024). As a result, researchers can better identify and validate drug candidates, optimize formulations, and forecast stability and performance, thereby significantly reducing development time and associated costs.

The field’s rapid evolution is driving a major shift in pharmaceutical R&D, moving away from reliance on empirical knowledge and manual experimentation toward a systematic, computation-centric methodology. This transformation not only enhances accuracy and reproducibility but also accelerates the translation of molecular discoveries into clinically viable therapies. As such, computational pharmaceutics stands at the forefront of modern pharmaceutical innovation, playing a pivotal role in shaping the future of formulation science and personalized medicine.

**FIGURE 5:**
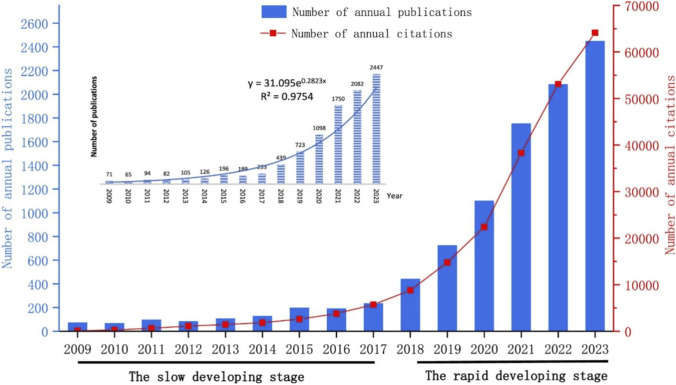
Annual number of publications and citations in the field of AI-aided drug discovery from 2009 to 2023. The bar chart (left axis, blue) shows the number of annual publications, while the line graph (right axis, red) depicts the number of annual citations. The trend indicates two distinct phases of development: a slow growth period from 2009 to 2017, followed by rapid expansion from 2018 to 2023. The data show exponential growth in both metrics, with publications reaching 2,447 and citations climbing to 64,137 in 2023. Data adapted from Wenshuo Jian et al. (2025).

However, Figure 5 visually presents the rapid growth in scientific literature related to artificial intelligence (AI) applications in pharmaceutical research over the past 2 decades. From 2000 to approximately 2014, the record count demonstrates a pattern of steady annual growth, reflecting the early integration of computational approaches in drug development. Starting around 2015, a marked upward trend is observed, with a significant surge in publications signaling the widespread adoption of AI technologies across the field.

Post-2018, the figure shows an explosive increase in publication volume, indicating accelerated research activity and heightened global interest in AI-driven pharmaceutics. The bar graph highlights a peak in record count in 2023, underscoring the contemporary relevance and momentum of this domain. This trend reflects the growing trust and reliance on AI and machine learning tools in transforming traditional pharmaceutical R&D practices into predictive, data-driven methodologies.

Recent trends in academic research underscore the accelerating growth of artificial intelligence (AI)-aided drug discovery. As illustrated in Figure 5, the number of annual publications in this field has exhibited a clear exponential growth pattern over the past 15 years. According to Wenshuo Jiang et al., the developmental trajectory of AI-driven pharmaceutical research can be divided into two distinct phases: a *slow development stage* from 2009 to 2017, and a *rapid development stage* from 2018 to 2023 (Jian et al., 2025).

During the early period (2009–2017), annual publications remained modest, gradually rising from just over 70 in 2009 to nearly 250 in 2017. This phase laid the groundwork for future breakthroughs. From 2018 onward, however, the field witnessed a dramatic increase in scientific output. The number of publications surpassed 1,000 in 2020 and exceeded 2,000 by 2022, reaching 2,447 in 2023 alone. This sharp rise reflects the growing recognition and adoption of AI technologies in pharmaceutical sciences.

A parallel trend is observed in citation metrics. Annual citations grew steadily, particularly from 2020 onward, where they ranged between 10,000 and 20,000 annually. In 2023, AI-aided drug discovery publications were cited 64,137 times, reflecting not only increased academic interest but also the field’s growing impact on the broader scientific community. Projections for 2024 suggest a continued upward trajectory in both publication output and citations, further cementing AI’s pivotal role in modern drug discovery pipelines.

However, emerging computational methodologies including structure-based virtual screening, data-driven machine learning (ML), and deep learning (DL) approaches are now widely utilized to predict the Absorption, Distribution, Metabolism, Excretion, and Toxicology (ADMET) properties, as well as pharmacokinetic (PK) profiles, of candidate compounds (Sadybekov et al., 2022); (Lalmuanawma et al., 2020); (Gordon et al., 2020); (Dhakal et al., 2022). These approaches leverage high-performance computing technologies such as cloud-based platforms and graphics processing units (GPUs), enabling the efficient processing of large-scale molecular datasets across multiple stages of the drug discovery pipeline. For example, deep learning models are capable of analyzing vast quantities of structural and biological activity data to predict ADMET properties, thereby facilitating the identification of compounds with favorable PK characteristics (Ferreira and Andricopulo, 2019; Jia et al., 2020).

Moreover, ML algorithms are increasingly applied in toxicity prediction, enabling the early identification of potentially harmful side effects. This not only reduces the risk of late-stage drug failure but also enhances safety profiling during preclinical evaluation (PotentialNet for Molecular Property Prediction, 2025), (Zeng et al., 2022; Tian et al., 2015; Raies and Bajic, 2016). As a result, computational chemistry has evolved from a supplementary role into a central component of modern drug discovery, with numerous computationally designed drug candidates progressing to clinical trial stages.

Computational pharmaceutics has demonstrated notable success in accelerating the development of treatments for a range of diseases, including cancers, viral infections, and neurodegenerative disorders. As computational tools continue to evolve, they are expected to play increasingly pivotal roles in hit identification, lead optimization, and multi-parameter optimization processes. The integration of these methods is anticipated to improve discovery success rates, reduce research and development (R&D) costs, and enable the creation of more effective and safer therapeutics. In parallel, the advancement of personalized medicine and precision healthcare is further amplifying the impact of computational pharmaceutics. By incorporating patient-specific genetic, epigenetic, and lifestyle data, these tools support the development of customized drug formulations tailored to individual biological profiles. Multi-omics data integration and big data analytics provide the foundation for precise prediction of therapeutic responses, offering a robust scientific basis for designing patient-centric treatment strategies. This paradigm shift positions computational pharmaceutics as a cornerstone in the realization of precision medicine, with significant implications for improving therapeutic outcomes and minimizing adverse drug reactions.

### 2.2 Industry 4.0 for pharmaceutical manufacturing

**FIGURE 6:**
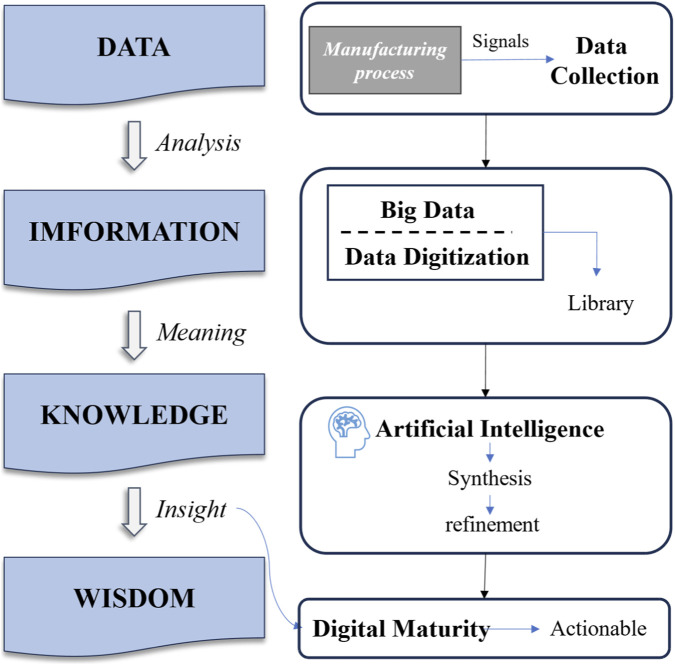
Data-to-wisdom hierarchy model illustrating the digital transformation of pharmaceutical manufacturing processes under Industry 4.0. The model traces the progression from raw data collection through data digitization and big data analytics to generate structured information. This information is further processed into actionable knowledge using intelligent technologies, ultimately culminating in wisdom strategic decision-making that enhances efficiency, quality, and sustainability in pharmaceutical production. The framework underscores the essential role of smart technologies in transforming data into value within modern manufacturing ecosystems.

Over the past two centuries, the pharmaceutical industry has undergone a profound transformation, evolving from the use of rudimentary herbal preparations to the development of complex and highly specialized pharmaceutical products and dosage forms. In recent years, pharmaceutical manufacturing technologies have continued to advance, catalyzed by the emergence of the Internet of Things (IoT), artificial intelligence (AI), robotics, and high-performance computing all of which are challenging traditional production paradigms and business models (Figure 6; Arden et al., 2021).

These technological innovations hold considerable promises for enhancing the agility, efficiency, flexibility, and overall quality of pharmaceutical manufacturing processes. Their continuous evolution has contributed to the rise of the industry 4.0 framework, which marks a new era in industrial advancement characterized by the convergence of smart technologies with conventional manufacturing systems (Folgado et al., 2024).

At its core, Industry 4.0 involves the integration of large-scale machine-to-machine (M2M) communication and IoT infrastructures. This facilitates a high degree of automation, real-time process monitoring, and intelligent diagnostics through interconnected devices capable of analyzing and resolving issues autonomously minimizing the need for human intervention (Sharma et al., 2023).

The implementation of Industry 4.0 technologies has fundamentally redefined manufacturing workflows, offering a multitude of benefits. These include increased production yields, enhanced operational safety, improved product consistency and quality, greater responsiveness to customer needs, elevated process agility, and reduced material waste. Collectively, these advancements position Industry 4.0 as a transformative force in modern pharmaceutical manufacturing, paving the way for smarter, safer, and more sustainable production models.

The transition to Industry 4.0 in pharmaceutical manufacturing is underpinned by the ability to transform raw data into actionable intelligence. Figure 6 describes the data-to-wisdom hierarchya foundational model that highlights how digital transformation enables smarter and more informed decision-making in pharmaceutical production.

This framework begins with the generation of raw data from various stages of the manufacturing process. Through data digitization and big data technologies, this raw input is converted into information, which is then processed and analyzed to generate meaningful knowledge. Advanced computational tools, such as AI and machine learning, are instrumental in this stage, facilitating the extraction of patterns, trends, and operational insights from large, complex datasets.

Subsequently, this knowledge forms the basis for achieving wisdom the ability to make strategic, informed decisions that enhance manufacturing efficiency, ensure product quality, and optimize resource utilization. The flow from data to wisdom represents a transformative journey, aligning with the principles of smart manufacturing and reinforcing the role of Industry 4.0 in driving evidence-based operations.

At the heart of the Industry 4.0 paradigm lies its capacity for powerful integration and intelligent automation. By embedding artificial intelligence (AI) into robotic systems, pharmaceutical manufacturing environments are increasingly capable of functioning autonomously, with minimal human intervention. These integrated autonomous robotic systems harness real-time data—both online and offline and synergize it with industrial production workflows and enterprise-level decision-making frameworks (Moore, 2019; Sharma et al., 2020). This convergence enables dynamic optimization of manufacturing operations, quality control, and resource management.

Furthermore, the seamless integration of internal and external data sources facilitates unprecedented capabilities in real-time monitoring, control, predictive analytics, and system responsiveness. Such capabilities allow pharmaceutical manufacturers to establish a fully digital, highly connected ecosystem, characterized by a well-controlled and intelligent manufacturing environment. This digital transformation not only improves operational efficiency and consistency but also supports adaptive and resilient production systems aligned with modern regulatory and market demands. Where available, quantitative metrics such as error reductions, throughput gains, energy savings, and downtime cuts are reported to differentiate laboratory demonstrations from implemented pilot-scale and GMP operations.

**FIGURE 7:**
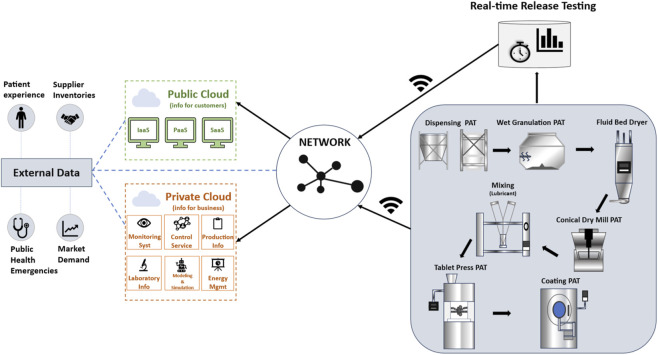
Smart manufacturing architecture in pharmaceutical production integrating external and internal data sources via public and private cloud infrastructure. The public cloud delivers customer-oriented services (IaaS, PaaS, SaaS), while the private cloud manages internal business operations including monitoring systems, laboratory information, production control, modeling and simulation, and energy management. On the production line, real-time process analytical technology (PAT) tools are implemented across each unit operation (e.g., granulation, drying, milling, tableting, and coating), facilitating continuous monitoring and control. The system enhances real-time responsiveness, predictive capability, and end-to-end digital coordination in pharmaceutical manufacturing.

Meanwhile, Figure 7 exemplifies the digital architecture underpinning smart pharmaceutical manufacturing systems, highlighting the integration of external and internal data through cloud-based infrastructure. In this configuration, external data sources including clinical input, market demand, and healthcare-related analytics are funneled into both public and private cloud environments. The public cloud supports customer-facing services via infrastructure-as-a-service (IaaS), platform-as-a-service (PaaS), and software-as-a-service (SaaS) models. In parallel, the private cloud facilitates business-critical functions such as monitoring systems, laboratory data management, production control, modeling and simulation, and energy management.

This dual-cloud architecture enables real-time, bi-directional communication and intelligent coordination across all units of pharmaceutical manufacturing. On the production floor, process analytical technology (PAT) tools are embedded throughout each stage from dispensing, wet granulation, and drying, to mixing, milling, tablet pressing, and coating. These PAT-enabled units generate continuous feedback loops for data-driven process optimization.

Crucially, this system design allows for real-time data collection, performance tracking, and predictive analytics, creating a closed-loop manufacturing environment. The seamless integration of AI and industrial internet of things (IIoT) components results in improved process control, faster decision-making, and reduced human error core principles of Industry 4.0 and the smart factory model.

### 2.3 AI technologies that can be deployed on pilot platforms

Methodologically, AI-enabled pilot platforms operate across four interconnected layers: (i) molecular and synthesis modeling, (ii) formulation and process modeling, (iii) real-time monitoring and control using PAT, and (iv) pilot-scale validation under quality-by-design frameworks. Throughout this review, each layer is illustrated with representative tools and case examples to demonstrate how computational methods translate into practical pilot-scale implementation.

#### 2.3.1 Algorithmic foundations of AI-Enabled pilot-scale pharmaceutics

Artificial intelligence applied to pilot-scale pharmaceutical platforms relies not on a single algorithmic paradigm, but on a coordinated hierarchy of models optimized for heterogeneous data, constrained process environments, and regulatory oversight. Unlike laboratory-scale AI demonstrations, pilot-scale systems must operate under limited data availability, process noise, equipment variability, and Good Manufacturing Practice (GMP) constraints, necessitating algorithmic choices that prioritize robustness, interpretability, and generalization.

In synthetic route design, modern computer-assisted synthesis planning (CASP) systems increasingly employ graph-based and transformer-based architectures rather than rule-based heuristics. Graph neural networks (GNNs) encode molecular structures as node–edge representations, enabling chemically meaningful learning of reaction centers and bond transformations, while transformer models treat reactions as sequence prediction tasks to capture long-range dependencies across multistep retrosynthetic pathways (Schwaller et al., 2019; Segler and Waller, 2017; Han et al., 2022b). These approaches outperform earlier expert systems by learning directly from large-scale reaction datasets and dynamically ranking pathways based on feasibility, yield, cost, and sustainability metrics.

For pilot-scale process monitoring and control, algorithm selection is driven by real-time deployment requirements. Hybrid chemometric–machine learning frameworks are commonly adopted, in which multivariate statistical models such as partial least squares (PLS) are used for spectral interpretation and moisture estimation, while tree-based ensemble models (e.g., LightGBM, XGBoost) handle nonlinear relationships between process variables and critical quality attributes (CQAs) (Roggo et al., 2007; Gorle et al., 2025). These models are favored over deep neural networks in many pilot environments because they provide faster inference, improved interpretability, and lower data requirements while maintaining high predictive accuracy.

Closed-loop control systems typically integrate these predictive models within model predictive control (MPC) frameworks, allowing proactive adjustment of critical process parameters while respecting operational and safety constraints. Increasingly, human-in-the-loop control strategies are adopted, in which AI-generated recommendations require operator confirmation prior to execution, aligning algorithmic autonomy with regulatory expectations for human oversight and accountability (U.S. Food and Drug Administration FDA, 2021), (European Medicines Agency EMA, 2024). Collectively, these algorithmic architectures reflect a shift from isolated AI tools toward systems-level intelligence that supports robust, interpretable, and compliant pilot-scale pharmaceutical manufacturing.

#### 2.3.2 Drug synthesis path planning

The development of computer-assisted synthesis planning (CASP) has played a transformative role in the evolution of drug synthesis. The origins of CASP can be traced back to the 1960s, when E. J. Corey and his team introduced LHASA (Logic and Heuristics Applied to Synthetic Analysis) (Corey, 1967), a pioneering rule-based system for retrosynthetic design (Corey and Wipke, 1969). LHASA is widely regarded as the foundational model upon which modern CASP technologies were built (Corey and Wipke, 1969). Prior to the 1980s, chemical reaction data were often recorded manually using index cards, making information retrieval inefficient and error prone. However, with the advancement of computer technologies, such manual systems have been largely replaced by powerful chemical databases such as SciFinder and Reaxys.

Contemporary CASP systems have achieved remarkable progress, enabled by the adoption of advanced algorithms including Convolutional Neural Networks (CNNs) (LeCun et al., 1989), Recurrent Neural Networks (RNNs) (Elman, 1990), Graph Neural Networks (GNNs) (A new model for learning, 2025), and Atom-Mapping (AM) techniques (ReactionMap: An Efficient Atom, 2025), (Wang et al., 2021b). These computational models assist chemists in identifying efficient, high-yielding, and environmentally friendly synthetic pathways for complex target molecules (Computer Assisted, 2025), (Jiang, 2012). In recent years, the integration of artificial intelligence particularly the Transformer architecture initially developed for natural language processing has further revolutionized CASP applications (Corey and Wipke, 1969).

One notable example is the work by Luhua Lai and colleagues, who developed a Transformer-based reaction prediction framework. Building upon this, they introduced *AutoSynRoute*, an automated synthesis route planning tool that utilizes single-step retrosynthesis, heuristic scoring, and Monte Carlo Tree Search (MCTS) strategies for multi-step optimization (Lin et al., 2020), (Coulom et al., 2006). This innovative system enhances the decision-making process in synthesis design, offering a scalable and intelligent solution for synthetic planning. Collectively, these developments illustrate the significant potential of AI-driven tools to automate and optimize drug synthesis pathways, marking a major advancement in modern pharmaceutical manufacturing.

**FIGURE 8:**
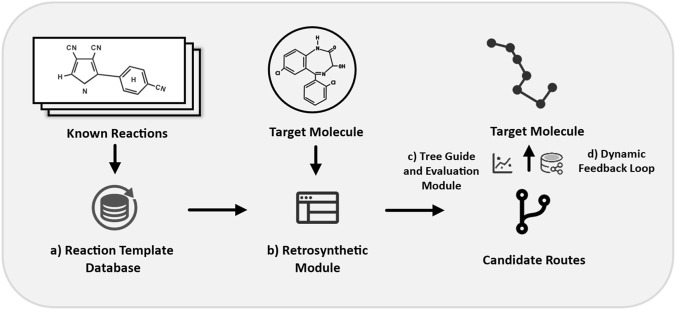
Schematic representation of AI-driven drug synthesis path planning. **(a)** A reaction template database serves as the foundation for known chemical transformations. **(b)** The retrosynthetic module decomposes the target molecule into possible precursors. **(c)** The tree guide and evaluation module generates and scores candidate routes based on heuristic and predictive metrics. **(d)** A dynamic feedback loop refines the synthetic plans through iterative learning and optimization, ultimately identifying feasible pathways for the target molecule.

Although the Figure 8 demonstrates the conceptual workflow of AI-assisted drug synthesis path planning using a computer-assisted synthesis planning (CASP). The process begins with a curated reaction template database (a), comprising known reactions and synthetic precedents. These templates provide the foundational rules for retrosynthetic decomposition. The retrosynthetic module (b) then analyzes a given target molecule by breaking it down into simpler precursors based on these reaction rules. This step generates a set of possible synthetic disconnections and intermediate structures. Next, the tree guide and evaluation module (c) construct and evaluates synthetic route trees by scoring candidate pathways based on metrics such as yield, step count, green chemistry principles, and cost-effectiveness. Through this step, multiple candidate synthesis routes are proposed. Finally, a dynamic feedback loop (d) allows iterative refinement of the synthetic pathways. By integrating feedback from predictive models and experimental validations, the system continuously improves the selection of routes, ultimately converging to an optimal or near-optimal synthesis plan for the target molecule.

This integrated workflow highlights the synergistic application of AI, retrosynthetic analysis, and heuristic evaluation in modern CASP systems, exemplifying the potential for intelligent automation in drug synthesis planning.

Recent advances in computer-assisted synthesis planning (CASP) have expanded its applicability far beyond the early rule-based systems such as LHASA and SECS (Simulation and Evaluation of Chemical Synthesis) (Wang et al., 2021c). While these pioneering methods were effective for guiding chemical synthesis in controlled laboratory environments, they were inherently limited by rigid, manually encoded heuristics. The integration of machine learning particularly deep learning and neural network architectures, has since transformed CASP into a far more flexible and autonomous tool for synthetic design (Pflüger and Glorius, 2020). Modern frameworks leverage advanced computational models such as neural networks and sequence-to-sequence (seq2seq) architectures to learn reaction rules from large-scale chemical datasets, eliminating the need for manual rule encoding. A notable example is the 3N-MCTS algorithm, developed by the group led by Mark Waller and Marwin Segler. This approach combines three neural networks with Monte Carlo Tree Search (MCTS) to autonomously extract and apply reaction rules through deep learning. The result is an intelligent system capable of performing efficient and accurate retrosynthetic analysis without human intervention (Segler et al., 2018).

Further progress has been demonstrated by Dong et al., who introduced *ReSynZ*, an algorithm inspired by the reinforcement learning strategy used in AlphaGo. This model significantly improves synthetic pathway search by optimizing both efficiency and accuracy in decision-making processes (Guo et al., 2024). In parallel, researchers at the University of Bayreuth proposed *ProtGPT2*, a novel generative framework built upon a stacked Transformer decoder. This system explores unexplored regions of the chemical space, generating drug-like molecules that demonstrate predictive stability and dynamic properties akin to naturally occurring sequences (Ferruz et al., 2022).

Collectively, these cutting-edge approaches represent a paradigm shift in synthesis planning, offering robust, scalable, and intelligent solutions that bridge cheminformatics, AI, and pharmaceutical research. These innovations not only accelerate the discovery of viable synthetic routes but also open new avenues for *de novo* drug design and precision formulation development.

**FIGURE 9:**
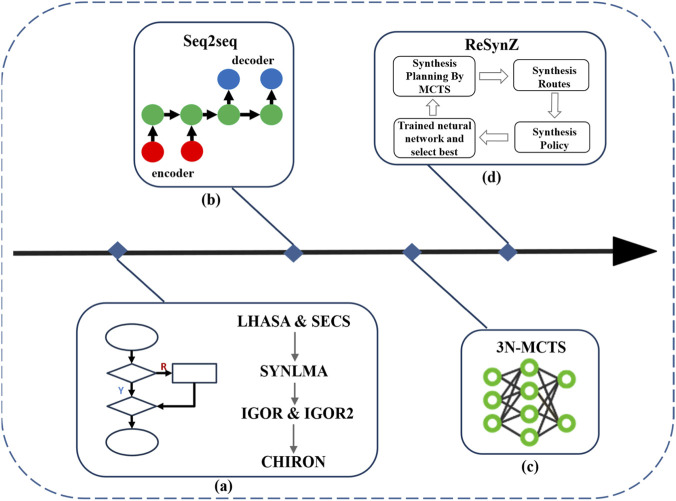
Evolution of computer-assisted synthesis planning (CASP) methodologies. **(a)** Rule-based systems such as LHASA and SECS laid the groundwork for synthetic planning, later evolving into IGOR, CHIRON, and other expert systems. **(b)** Sequence-to-sequence (Seq2seq) neural networks enabled data-driven retrosynthesis using encoder-decoder architectures. **(c)** The 3N-MCTS model integrates multiple neural networks with Monte Carlo Tree Search for autonomous synthesis planning. **(d)** ReSynZ leverages reinforcement learning and trained neural networks to optimize synthesis routes and policies. The figure outlines the progression from manual expert systems to advanced AI-guided synthesis technologies.

The evolution of computer-assisted synthesis planning (CASP) reflects a remarkable trajectory from early rule-based systems to advanced, AI-driven platforms, as shown in Figure 9. Initially, synthesis planning relied on expert systems such as LHASA and SECS, which utilized manually curated reaction rules to propose retrosynthetic pathways. These foundational tools were subsequently expanded into more sophisticated platforms, including SYNLMA, IGOR, IGOR2, and CHIRON, which offered enhanced capabilities for handling increasingly complex chemical structures and synthetic logic. However, their reliance on fixed rules sets limited adaptability and scalability across diverse reaction spaces. With the advent of machine learning, particularly deep learning, CASP systems began integrating data-driven models such as sequence-to-sequence (Seq2seq) architectures. These encoder-decoder neural networks enabled the prediction of synthetic steps directly from molecular data, thereby bypassing the need for explicit rule definition. This transition marked a critical step toward automation in synthetic route planning. The development of hybrid frameworks such as 3N-MCTS further advanced this shift. By integrating three distinct neural networks with Monte Carlo Tree Search (MCTS), 3N-MCTS autonomously extracted and applied synthetic logic learned from large datasets, dramatically improving both the accuracy and efficiency of retrosynthetic analysis.

More recently, reinforcement learning has been employed to develop dynamic, policy-driven CASP models such as ReSynZ. This framework combines a trained neural network with MCTS to iteratively generate and evaluate synthesis routes based on optimized reward strategies. ReSynZ exemplifies the movement toward intelligent systems capable of not only planning chemical syntheses but also refining their decision-making processes over time. Collectively, these advancements underscore the transformative role of artificial intelligence in reshaping the landscape of chemical synthesis planning.

#### 2.3.3 AI-assisted process control in continuous granulation

This section presents a validated pilot-scale implementation conducted under near-GMP conditions, included to replace conceptual-only illustrations and to demonstrate empirically how AI-enabled control strategies operate in real pilot environments. To strengthen the practical relevance of AI-enabled process control, a worked pilot-scale case was incorporated to replace the previous conceptual-only schematic. This example illustrates how Quality by Design (QbD), Process Analytical Technology (PAT), and predictive modeling operate in a continuous granulation environment representative of pilot and near-GMP conditions (Destro et al., 2024). A twin-screw wet granulation line was used, followed by a segmented fluid-bed dryer, mill, and blender. The formulation consisted of a lactose–microcrystalline cellulose blend containing a model active pharmaceutical ingredient at 5% (Celikovic et al., 2023). Real-time data streams included near-infrared (NIR) spectra at 1-s resolution, torque and barrel temperature signals, granule size distribution from on-line focused beam reflectance measurement (FBRM), and moisture predictions computed using a partial least squares (PLS) model (RMSEP = 0.48) (Muhaimin et al., 2021), (Záhonyi et al., 2025).

On this basis, to achieve real-time prediction and modeling of critical quality attributes (CQAs), researchers usually adopt a hybrid modeling strategy based on PAT data, combining chemometrics methods with machine learning models to characterize the behavior of complex nonlinear processes. For instance, Celikovic et al. constructed a prediction model based on local linear model tree (LoLiMoT) on the ConsiGma-25 continuous production line by using NIR, FBRM and process parameter signals, which was used to estimate CQAs such as particle size distribution, API content and moisture content online (Celikovic et al., 2023). So as to achieve dynamic characterization of the product quality status. Meanwhile, Vega-Zambrano et al. proposed a data-driven model based on Dynamic Mode Decomposition control (DMDc) to describe the dynamic behavior of the twin-screw wet granulation process and achieve high-precision prediction of the median particle size (d50), with a goodness of fit on the test dataset reaching *R* ^2^ > 0.93 (Vega-Zambrano et al., 2025).

**FIGURE 10:**
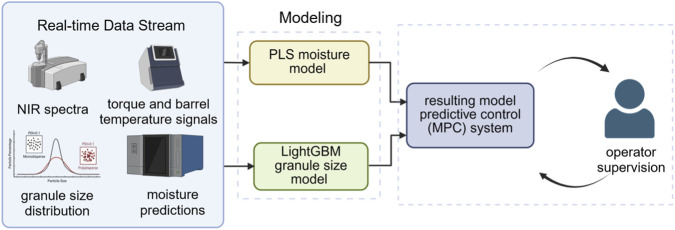
AI-assisted predictive control framework implemented on a pilot-scale continuous granulation line. Real-time data streams including NIR spectra, torque and temperature signals, granule size distribution, and moisture predictions serve as inputs for two predictive models: a partial least squares (PLS) moisture model and a LightGBM granule-size model. These models feed a human-in-the-loop model predictive control (MPC) system, which recommends adjustments to key process parameters in the pilot equipment. The deployment resulted in measurable performance improvements, including a 32% reduction in moisture variability, a 41% reduction in out-of-spec granule-size events, a 67% decrease in torque-related line stoppages, an 8.6% increase in yield, and an extension of screw-bearing service intervals from 90 to 230 h. The schematic illustrates the integrated data flow, modeling components, operator-supervised MPC loop, and key performance outcomes achieved during pilot operation.

Based on the above prediction model, it was further integrated into the Model Predictive Control (MPC) framework to construct a closed-loop quality control system for the continuous granulation process. Previous studies have shown that MPC based on data-driven models can utilize the model prediction results to adjust key process parameters such as screw speed and liquid feeding rate in real time, thereby maintaining the stable operation of key quality attributes such as particle size and water content within the target range (Celikovic et al., 2023; Celikovic et al., 2024). In practical applications, control actions typically adopt a human-machine collaborative approach - that is, before performing critical adjustments, the operator needs to review and confirm to meet the GMP regulatory requirements for automated adjustments (Celikovic et al., 2024). All chemometric and machine-learning models were validated using FDA-aligned criteria, including PCA residual diagnostics, Hotelling’s T^2^ control limits, bias testing, and RMSEP evaluation, reinforcing the applicability of this approach to regulated manufacturing environments. Figure 10 show the data flow, modeling architecture, human-in-the-loop MPC logic, and pilot-scale KPI improvements for this continuous granulation case.

#### 2.3.4 Process monitoring and predictive maintenance

The application of artificial intelligence (AI) in industrial process monitoring and control has been steadily progressing since the mid-1980s (Rich and Venkatasubramanian, 1987). However, it is only in recent decades—driven by advancements in computational capabilities—that AI has demonstrated extensive real-world utility (Nagy et al., 2019; Sharma et al., 2024). Today, AI technologies are highly mature and are increasingly employed in areas such as process optimization, predictive analytics, and asset management, reinforcing their pivotal role in industrial automation and intelligent manufacturing.

Recent studies provide quantitative evidence demonstrating the measurable impact of AI and PAT in pharmaceutical manufacturing. For example, integrating Raman and NIR-based PAT in continuous granulation has reduced end-product variability by 20%–35% and decreased batch failure rates by approximately 25%. AI-enabled predictive maintenance systems have achieved a 30%–50% reduction in unplanned downtime in validated pilot-scale units, while improving equipment lifetime by roughly 15%. In contrast, laboratory-scale demonstrations often report larger performance improvements (up to 60%–70%), but these results typically reflect controlled experimental conditions rather than GMP-relevant variability. To maintain clarity, this review distinguishes between laboratory-level proofs of concept and validated pilot/GMP outcomes, highlighting the practical performance metrics achievable in real operational environments.

In the context of pilot-scale pharmaceutical production, AI enables the rapid and precise analysis of large datasets, facilitating real-time, data-informed decision-making. By leveraging AI models, interdisciplinary teams can efficiently manage and interpret complex datasets. For instance, Li et al. (2022) employed an AI-integrated multi-omics approach for glycomics data analysis between 2017 and 2021, showcasing the capacity of AI to streamline data interpretation in biomedical research.

The Quality by Design (QbD) framework plays a critical role in pharmaceutical quality assurance. As a systematic risk-based methodology, QbD emphasizes predefined objectives, thorough process understanding, and robust control strategies rooted in sound scientific principles and risk management (ICH Q8, 2024). In the AI era, integrating QbD with Process Analytical Technology (PAT) has substantially improved quality control and productivity across pharmaceutical workflows (Zhang and Mao, 2017; Lee et al., 2022). Originally introduced in the U.S. FDA’s 2004 guidance, PAT encourages the adoption of innovative technologies for real-time product quality monitoring (C. for D. E. and Research, 2025).

PAT systems incorporate a variety of analytical techniques such as Raman spectroscopy, UV-Vis, Nuclear Magnetic Resonance (NMR), Near Infrared Spectroscopy (NIR), Focused Beam Reflectance Measurement (FBRM), NanoTemperature Measurement (NTM), and Tunable Diode Laser Absorption Spectroscopy (TDLAS) to enable real-time tracking of Critical Quality Attributes (CQAs) and Critical Process Parameters (CPPs) during manufacturing (Troup and Georgakis, 2013; Neugebauer et al., 2024). These technologies are often complemented by advanced control strategies like Model Predictive Control (MPC) and Proportional-Integral-Derivative (PID) controllers. MPC applies predictive modeling to optimize current control actions, while PID regulators use feedback loops to stabilize CQAs, thereby maintaining consistent product quality. Currently, PID controllers remain widely used in industrial settings (Singh et al., 2014; Bhaskar et al., 2017).

In parallel, the pharmaceutical industry is witnessing a shift from traditional regression models to AI-driven machine learning and deep learning algorithms for enhanced analytical performance. Modern techniques include Convolutional Neural Networks (CNNs), Recurrent Neural Networks (RNNs), Generative Adversarial Networks (GANs), and ensemble methods such as Random Forests, Gradient Boosted Decision Trees (GBDTs), XGBoost, LightGBM, and CatBoost (Bhaskar et al., 2017; Uraikul et al., 2007). These algorithms enhance model robustness, accuracy, and generalization, especially when applied to high-dimensional, nonlinear datasets.

An example of AI integration in industrial applications is the automated chemical processing system developed by Granda et al. (2018), which combines online spectroscopy, real-time analytics, and feedback loops (Vaswani et al., 2017). Their system uses Support Vector Machine (SVM) and Linear Discriminant Analysis (LDA) models to predict reactant reactivity and evaluate outcomes dynamically. This integration exemplifies the transformative potential of AI in enhancing manufacturing efficiency and process control.

Despite these advancements, challenges remain. A key limitation is the generalizability of AI models across varying production environments, compounded by issues related to data quality, volume, and accessibility. Therefore, future research must prioritize the development of more robust algorithms and strategies to enhance data utilization efficiency and model adaptability.

AI also plays a transformative role in optimizing Hot Melt Extrusion (HME) processes. Studies have integrated PAT tools (e.g., NIR, Raman, UV-Vis) with machine learning models to achieve real-time monitoring and control of the HME process (Munir et al., 2021; Gnoth et al., 2007). An ideal PAT system can continuously monitor critical quality parameters and adjust production conditions to ensure final product integrity.

Moreover, the data generated through PAT-based monitoring systems serve as foundational inputs for Predictive Maintenance (PdM). PdM leverages AI algorithms especially machine learning and deep learning models to analyze complex patterns and forecast equipment failures, thus minimizing unplanned downtime and improving process reliability (Kim and Choi, 2021; You and Meng, 2012). By enabling early fault detection and informed maintenance scheduling, PdM contributes to cost reduction and sustainable production practices.

While the integration of QbD, PAT, and PdM into AI-enabled platforms offers substantial promise, it also brings forth several challenges, such as data integration, algorithmic interpretability, and the need for comprehensive process understanding. Addressing these limitations requires more sophisticated AI techniques and tighter integration across these systems. Ultimately, future advancements should focus on the synergistic incorporation of QbD, PAT, PdM, and AI to enable intelligent, adaptive automation in pharmaceutical manufacturing.

**FIGURE 11:**
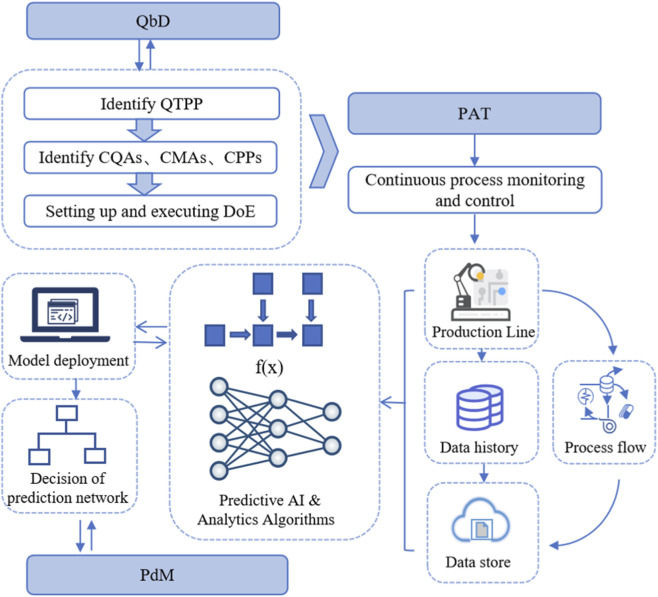
Schematic representation of an AI-integrated pharmaceutical production framework combining QbD, PAT, and PdM. The diagram illustrates the transition from QbD-defined parameters to PAT-enabled real-time monitoring, feeding into AI-driven predictive models for production optimization and maintenance. This interconnected system ensures continuous quality assurance and predictive control within a digitalized manufacturing environment.

However, Figure 11 describes the integration of Quality by Design (QbD), Process Analytical Technology (PAT), and Predictive Maintenance (PdM) within an AI-driven pharmaceutical manufacturing ecosystem. The framework begins with the QbD process, where Critical Quality Attributes (CQAs), Critical Material Attributes (CMAs), and Critical Process Parameters (CPPs) are defined based on Quality Target Product Profile (QTPP). These elements inform the experimental design (DoE) and feed into the PAT system for real-time process monitoring and control. PAT collects continuous data from the production line, encompassing process flows, data history, and data repositories. These data streams are then analyzed using predictive AI and analytics algorithms, which generate real-time insights and decisions for system optimization. Trained models are deployed into production to facilitate decision-making, enabling automated responses to quality deviations or system inefficiencies.

This predictive modeling loop also supports PdM by identifying early signs of equipment wear or failure, thereby minimizing downtime and enhancing operational efficiency. The seamless flow of information between QbD, PAT, and PdM highlights the power of AI in enabling intelligent, adaptive, and quality-focused pharmaceutical manufacturing.

#### 2.3.5 Artificial intelligence-driven industrial production and digital twins

Since the 1970s, when Deming et al. pioneered the first computer-program-controlled automated chemical laboratory system, multi-step automated drug synthesis has remained a long-standing goal within the pharmaceutical industry (Deming and Pardue, 1971; Wang et al., 2023a). Automated chemistry leverages modular systems composed of standard physical operations such as liquid handling robots, robotic transfer mechanisms for plates or sample vials, and computer-controlled heaters and shakers to significantly reduce the manual workload of laboratory personnel (Cao et al., 2021). These systems integrate continuous flow chemistry with process analytical technologies and robotic automation to replace human operators with fully automated modules capable of executing batch reactions in a controlled environment.

At the core of modern synthetic automation lies the computer-assisted synthesis planning (CASP) algorithm, which guides the automation workflow encompassing reagent storage, reaction execution, purification, and real-time analytical modules. Operated with high precision by robotic arms and monitored continuously via feedback from integrated detection systems, such platforms facilitate end-to-end automation of compound synthesis and data acquisition (Wang et al., 2020).

A landmark example is the modular robotic system “Chemputer” developed by Steiner et al., which employs a chemical programming language to convert synthesis protocols into low-level instructions executed by robotic hardware. This platform automates key synthesis steps—including reaction initiation, fluid transfer, separation, and purification—and was successfully used to synthesize three pharmaceutical compounds (benzylamine hydrochloride, rufinamide, and sildenafil) with yields comparable to those from traditional manual methods. Notably, this digital encoding of synthetic procedures offers the ability to publish, version-control, and port protocols across different hardware platforms, thereby improving reproducibility, safety, and collaborative workflows in chemical synthesis (Steiner et al., 2019).

Coley et al. further advanced the field with an automated and scalable synthesis platform integrating AI-based planning with robotic execution. Their system demonstrated successful synthesis of 15 drug-related small molecules including aspirin, lidocaine, (S)-warfarin, and Safran amide highlighting the platform’s capacity to reduce manual labor while enhancing synthesis efficiency. This work represents a significant step toward fully autonomous chemical synthesis and underscores the transformative role of AI and automation in accelerating innovation in the field (Neugebauer et al., 2024; Coley et al., 2019; Neugebauer et al., 2024; Breen et al., 2021).

The emergence of machine learning (ML) algorithms has catalyzed advances in automated synthesis. Various ML paradigms including supervised learning, unsupervised learning, and reinforcement learning are increasingly being applied across domains such as medicine, chemistry, and robotics (Wang et al., 2023b). Representation learning, which extracts salient features from high-dimensional molecular data, has proven essential for capturing subtle structural patterns in complex chemical reactions. Through architectures such as convolutional neural networks (CNNs) and recurrent neural networks (RNNs), representation learning enhances tasks like reaction prediction, property analysis, and catalyst design (Bengio et al., 2013; Zhu et al., 2023).

Additionally, transfer learning enables models trained in one domain to adapt to new tasks with minimal labeled data, accelerating the discovery of novel synthetic pathways. In chemical synthesis, this allows knowledge from established reaction datasets to be leveraged for identifying and optimizing new reaction classes, thereby streamlining the exploration of chemical space.

**FIGURE 12:**
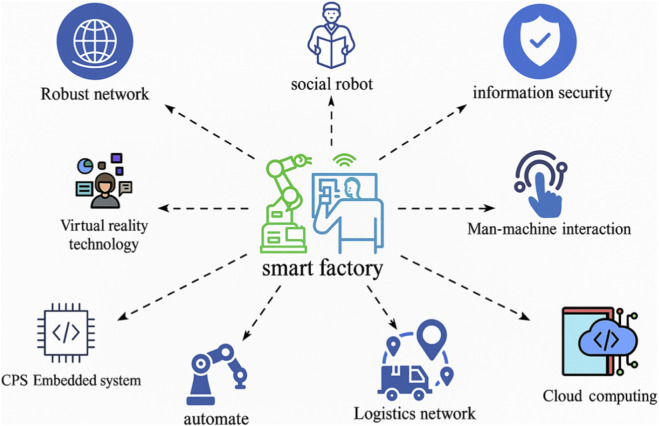
Smart factory ecosystem and its enabling technologies. The central smart factory is interconnected with a range of digital technologies including social robots, cybersecurity, cloud computing, CPS-embedded systems, virtual reality, and automated logistics networks. This integration enhances manufacturing flexibility, efficiency, and sustainability within Industry 4.0 environments.

Moreover Figure 12 presents an integrated overview of the smart factory ecosystem, highlighting the diverse technological components that collectively enable intelligent, autonomous, and highly adaptable pharmaceutical manufacturing under the Industry 4.0 paradigm. At the core of this ecosystem lies the smart factory, which serves as a digitally connected and self-optimizing production environment. The factory’s operations are empowered by cyber-physical systems (CPS), cloud computing, and embedded automation, which together support real-time monitoring, control, and decision-making. A robust network infrastructure facilitates seamless communication between devices and systems, ensuring data continuity and operational efficiency.

Surrounding this nucleus are various enabling technologies that contribute to the smart factory’s functionality. These include social robots capable of human-like interactions, which enhance man-machine collaboration, and intuitive man-machine interfaces that allow operators to effectively supervise and intervene when necessary. Information security frameworks are essential to safeguard sensitive industrial data against cyber threats, thereby maintaining system integrity. Additionally, the incorporation of virtual reality technologies provides immersive simulation environments for training, design validation, and predictive maintenance planning.

Cloud-based infrastructure plays a pivotal role by offering scalable storage and computational capabilities for data analytics and AI-driven optimization. Logistics networks and automated systems streamline supply chain operations, reducing delays and enhancing adaptability to production demands. Collectively, these technologies foster a highly integrated, efficient, and secure manufacturing environment that not only improves production quality and scalability but also aligns with the sustainable and human-centric goals of Industry 5.0.

#### 2.3.6 Data sources and model validation

The empirical examples and performance metrics discussed in this review are derived from aggregated pilot-scale studies and validated industrial case reports. Owing to intellectual-property constraints and GMP confidentiality, raw manufacturing datasets are not publicly released; model validation therefore follows the protocol defined in the FDA PAT Framework—“A Framework for Innovative Pharmaceutical Development, Manufacturing, and Quality Assurance” nd the ICH Q8 (R2) Pharmaceutical Development guideline (U.S. Food and Drug Administration, 2004), (International Conference on Harmonisation, 2014).

Model performance and reliability are assessed with FDA- and EMA-consistent criteria: root-mean-square error of prediction (RMSEP), bias testing, residual diagnostics, Hotelling’s T^2^ control limits (multivariate statistical process control), and external validation on independent pilot runs. These procedures conform to the statistical-quality-control methods detailed in Montgomery (Rayyad et al., 2023), (Montgomery, 2019). For machine-learning models deployed in process control or predictive maintenance, emphasis is placed on stability, generalisation across the design space, and interpretability rather than on maximal accuracy alone. This validation-centred approach ensures that all AI-enabled methodologies presented here are reproducible, auditable, and acceptable for regulated pharmaceutical manufacturing environments.

Despite the demonstrated benefits of AI-enabled pilot platforms, their translation into routine pharmaceutical manufacturing faces significant real-world challenges related to regulatory compliance, ethical deployment, and operational governance. Regulatory agencies continue to emphasize model transparency, traceability, and human accountability, which limits the adoption of black-box algorithms in GMP environments. AI models used for process control, predictive maintenance, or real-time release testing must be explainable, auditable, and validated across operating ranges, placing constraints on purely data-driven optimization approaches.

Ethical and governance considerations further complicate deployment, particularly with respect to data integrity, cybersecurity, and responsibility allocation between automated systems and human operators. Increasing reliance on interconnected digital infrastructure introduces vulnerabilities related to data manipulation, algorithmic drift, and unauthorized system access, necessitating robust cybersecurity frameworks and lifecycle model management. Moreover, the use of AI in regulated decision-making raises questions regarding accountability, bias propagation, and long-term model reliability. Addressing these challenges requires not only technical innovation, but also harmonized regulatory guidance, standardized validation strategies, and human-in-the-loop operational models that preserve oversight while enabling digital transformation.

## 3 Future research directions

As the pharmaceutical industry embraces digital transformation, a growing convergence of artificial intelligence (AI), advanced computational pharmaceutics, and smart manufacturing technologies are redefining traditional approaches to small molecule drug development. While recent advances have demonstrated significant promise in accelerating drug discovery, enhancing pilot-scale validation, and optimizing manufacturing processes, several critical areas demand sustained research to unlock the full potential of these technologies. This section outlines the future research directions essential for ensuring robust, intelligent, and sustainable drug development pipelines.

### 3.1 enhancing multi-scale integration of AI across the drug lifecycle

The integration of AI into the drug development pipeline has shown substantial benefits in discrete phases, such as synthesis prediction, process optimization, and quality control. However, a major challenge remains in developing multi-scale AI frameworks that operate seamlessly from early discovery through to commercial-scale production. Future research must explore end-to-end AI architectures that integrate molecular design tools, predictive synthesis planning, pilot-scale validation, and real-time manufacturing analytics into a cohesive system. Such frameworks should facilitate feedback loops between silico models and experimental systems, allowing iterative refinement of predictions based on real-world performance.

Furthermore, the integration of AI models across heterogeneous datasets including molecular structures, reaction kinetics, clinical outcomes, and manufacturing metrics requires the development of advanced data fusion techniques. Multi-modal AI architectures, such as transformer-based systems and hybrid neural-symbolic models, could help bridge the gap between low-level chemical descriptors and high-level process outcomes. This will support a holistic understanding of molecule behavior across scales and foster more informed decision-making throughout the drug lifecycle.

### 3.2 Strengthening the digital twin ecosystem for pharmaceutical production

The Digital Twin paradigm offers a powerful tool for real-time monitoring, simulation, and optimization of pharmaceutical production systems. However, existing digital twins are often limited in scope and fail to fully capture the dynamic interactions among equipment, raw materials, environmental conditions, and human operations. Future research must focus on building more comprehensive and modular digital twins that accurately reflect the multi-dimensional nature of pilot and industrial production systems.

To achieve this, domain-specific ontologies and semantic models are needed to ensure the interoperability of digital twin components across platforms and vendors. Furthermore, real-time data acquisition technologies including smart sensors, PAT (Process Analytical Technology), and IoT-enabled devices must be further developed and integrated into digital twins to ensure fidelity and responsiveness. Research should also explore the use of reinforcement learning and predictive maintenance algorithms within digital twins to enable proactive process adjustments, minimize downtime, and prevent quality deviations before they occur.

### 3.3 Advancing green chemistry and sustainable manufacturing

The pilot-scale integration of green chemistry principles, including biocatalysis, solvent substitution, and atom-economical reactions, has shown potential to improve both environmental sustainability and process efficiency. Yet, there remains a critical need for scalable and generalizable green manufacturing strategies that can be deployed across diverse chemical entities and production environments.

Future research should prioritize the development of eco-friendly synthetic pathways, supported by machine learning-guided route design. Special attention should be given to the integration of life cycle assessment (LCA) metrics into AI-based synthesis planning tools, enabling chemists to balance efficiency, cost, and environmental impact during the design phase. Moreover, investment in catalyst design and enzyme engineering, informed by generative AI and structural bioinformatics, could pave the way for cleaner, more selective reactions applicable to industrial contexts.

### 3.4 Personalized drug manufacturing and micro-factory platforms

As precision medicine and personalized healthcare continue to evolve, future drug production systems must adapt to support small-batch, patient-specific formulations. This will require the development of flexible manufacturing technologies, including modular micro-factories, that can rapidly produce high-quality drug products tailored to individual needs.

Research should focus on designing compact, reconfigurable manufacturing units embedded with AI-driven control systems and capable of autonomously adjusting to different molecular formulations and production scales. The application of continuous manufacturing principles in these micro-factories could further improve throughput and reproducibility. Additionally, AI-guided quality control systems, using advanced spectroscopic tools and chemometric algorithms, will be vital in ensuring that customized medications meet stringent safety and efficacy standards.

### 3.5 Expanding the role of computational pharmaceutics in predictive formulation

Computational pharmaceutics is poised to become a central pillar in rational drug formulation design. However, its current limitations in predicting complex physical behaviors such as polymorphism, dissolution, and stability warrant further exploration. Future research should aim to integrate multi-physics simulations with molecular modeling and data-driven approaches to capture the full spectrum of formulation performance.

Moreover, AI models that predict ADMET properties, pharmacokinetics, and drug-excipient interactions must be validated across diverse compound libraries to ensure generalizability. The establishment of open-access benchmark datasets and standardized evaluation metrics will be crucial for comparing and validating predictive algorithms across research groups. Future work could also focus on generative AI tools capable of suggesting novel excipient combinations and delivery systems tailored to specific therapeutic needs or patient profiles.

### 3.6 Human-machine collaboration and industry 5.0 transition

As the pharmaceutical industry transitions from Industry 4.0 to Industry 5.0, the focus will shift from full automation to human-centric innovation. This includes creating intelligent systems that not only assist but also collaborate with human experts in decision-making. Future research should investigate explainable AI (XAI) approaches that allow domain experts to understand, validate, and refine model outputs.

In addition, new human-machine interface (HMI) technologies, such as augmented reality (AR) and virtual reality (VR), offer immersive ways to interact with smart factory systems and could facilitate better training, troubleshooting, and process visualization. By fostering intuitive interaction between operators and AI systems, these technologies can enhance trust, improve safety, and accelerate technology adoption on the shop floor.

### 3.7 Standardization, data governance, and regulatory alignment

Despite the growing integration of AI and digital systems into pharmaceutical manufacturing, standardization and regulatory alignment remain significant bottlenecks. The future success of smart pharmaceutical ecosystems will depend on the development of interoperable data standards, secure data sharing frameworks, and transparent documentation of AI model behavior.

Future research must engage with regulatory bodies to develop adaptive guidelines and validation protocols for AI-driven systems. This includes the use of digital twins in Good Manufacturing Practice (GMP) environments, model risk management strategies, and compliance-aware AI design. Establishing trustworthy AI principles in pharmaceutical contexts will be key to widespread adoption and cross-border collaboration.

Simultaneously, data governance policies must address concerns around data ownership, privacy, and security, particularly when leveraging cloud platforms and multi-stakeholder ecosystems. Research on blockchain-based data provenance, federated learning, and secure multi-party computation can support data integrity while enabling collaborative innovation.

### 3.8 Bridging educational and talent gaps in AI-Driven pharmaceutics

The deployment of advanced technologies across the pharmaceutical value chain has outpaced the development of a skilled workforce trained in both life sciences and computational disciplines. Future research must support the creation of interdisciplinary education programs that merge pharmacy, chemistry, engineering, computer science, and data analytics.

Furthermore, new simulation-based and virtual training platforms should be developed to prepare technicians, scientists, and regulatory professionals for AI-augmented production environments. Research could also explore AI-enabled training assistants, capable of offering real-time guidance and feedback in lab and manufacturing settings, thereby accelerating onboarding and skill development.

In summary, the intersection of artificial intelligence, digital twin technologies, continuous manufacturing, and computational pharmaceutics represents a profound opportunity to revolutionize the development and production of small molecule drugs. However, to fully harness this potential, future research must address critical gaps in interoperability, model generalization, green chemistry, personalized production, and human-machine collaboration. By doing so, the pharmaceutical industry can transition toward a more agile, intelligent, and sustainable paradigm one that not only accelerates innovation but also ensures equitable access to high-quality therapeutics worldwide.

## 4 Conclusion

Artificial intelligence–enabled pilot-scale platforms are increasingly reshaping small molecule drug development by transforming traditionally empirical and fragmented workflows into integrated, data-driven systems. This review highlights how advances in computational pharmaceutics, AI-assisted synthesis planning, real-time process monitoring, and predictive control are strengthening the critical transition from laboratory discovery to scalable manufacturing. Evidence from validated pilot-scale implementations demonstrates that these technologies can improve process robustness, reduce variability, and shorten development timelines while supporting regulatory-aligned decision-making.

Importantly, the maturity of AI applications varies across the drug development pipeline. Computational methods for route design, formulation prediction, and process monitoring have reached a level of practical deployment under pilot and near-GMP conditions, whereas fully autonomous manufacturing and large-scale digital twin orchestration remain emerging capabilities. Successful implementation increasingly depends not only on algorithmic performance, but also on interpretability, human-in-the-loop control, and alignment with quality-by-design and process analytical technology frameworks.

This review also underscores that regulatory compliance, data governance, and system transparency represent central constraints on widespread industrial adoption. Ensuring explainable models, robust validation strategies, and secure data infrastructures is essential for translating AI innovations into regulated pharmaceutical environments. As the field advances toward Industry 5.0 paradigms, the emphasis is shifting from automation alone to human–machine collaboration, sustainability, and resilient manufacturing ecosystems.

Overall, AI-driven pilot platforms represent a critical enabling layer for the next-generation of small molecule drug development. When integrated thoughtfully with existing pharmaceutical quality systems and regulatory expectations, these platforms offer a realistic and impactful pathway for accelerating innovation while maintaining safety, quality, and compliance.

## References

[B1] A new model for learning (2025). A new model for learning in graph domains. IEEE Conference Publication | IEEE Xplore.” Accessed: April. 08, 2025. Available online at: https://ieeexplore.ieee.org/document/1555942

[B3] Anthwal A. Uniyal A. Gairolla J. Singh R. Gehlot A. Abbas M. (2024). Industry 4.0 technologies adoption for digital transition in drug discovery and development: a review. J. Industrial Inf. Integration 38, 100562. 10.1016/j.jii.2024.100562

[B4] Arden N. S. Fisher A. C. Tyner K. Yu L. X. Lee S. L. Kopcha M. (2021). Industry 4.0 for pharmaceutical manufacturing: preparing for the smart factories of the future. Int. J. Pharm. 602, 120554. 10.1016/j.ijpharm.2021.120554 33794326

[B5] Beck H. Härter M. Haß B. Schmeck C. Baerfacker L. (2022). Small molecules and their impact in drug discovery: a perspective on the occasion of the 125th anniversary of the bayer chemical research laboratory. Drug Discov. Today 27 (6), 1560–1574. 10.1016/j.drudis.2022.02.015 35202802

[B6] Bengio Y. Courville A. Vincent P. (2013). Representation learning: a review and new perspectives. IEEE Trans. Pattern Anal. Mach. Intell. 35 (8), 1798–1828. 10.1109/TPAMI.2013.50 23787338

[B7] Bhaskar A. Barros F. N. Singh R. (2017). Development and implementation of an advanced model predictive control system into continuous pharmaceutical tablet compaction process. Int. J. Pharm. 534 (1), 159–178. 10.1016/j.ijpharm.2017.10.003 28986318

[B8] Breen C. P. Nambiar A. M. K. Jamison T. F. Jensen K. F. (2021). Ready, set, flow! automated continuous synthesis and optimization. Trends Chem. 3 (5), 373–386. 10.1016/j.trechm.2021.02.005

[B9] C. for D. E. and Research (2025). “PAT — a framework for innovative pharmaceutical development, Manufacturing, and quality assurance.” Accessed: June. 11, 2024. Available online at: https://www.fda.gov/regulatory-information/search-fda-guidance-documents/pat-framework-innovative-pharmaceutical-development-manufacturing-and-quality-assurance

[B10] Cao L. Russo D. Lapkin A. A. (2021). Automated robotic platforms in design and development of formulations. AIChE J. 67 (5), e17248. 10.1002/aic.17248

[B11] Celikovic S. Poms J. Khinast J. Horn M. Rehrl J. (2023). Control oriented modeling of twin-screw granulation in the ConsiGmaTM-25 production plant. Int. J. Pharm. 641, 123038. 10.1016/j.ijpharm.2023.123038 37182794

[B12] Celikovic S. Poms J. Khinast J. Horn M. Rehrl J. (2024). Development and application of control concepts for twin-screw wet granulation in the ConsiGmaTM-25: part 2 granule size. Int. J. Pharm. 657, 124125. 10.1016/j.ijpharm.2024.124125 38631483

[B13] Coelho P. Bessa C. Landeck J. Silva C. (2023). Industry 5.0: the arising of a concept. Procedia Comput. Sci. 217, 1137–1144. 10.1016/j.procs.2022.12.127

[B14] Coley C. W. Thomas D. A. Lummiss J. A. M. Jaworski J. N. Breen C. P. Schultz V. (2019). A robotic platform for flow synthesis of organic compounds informed by AI planning. Science 365 (6453), eaax1566. 10.1126/science.aax1566 31395756

[B15] Computer Assisted (2025). Computer‐assisted planning of organic syntheses: the second generation of programs - ihlenfeldt - 1996. Angewandte Chemie International Edition in English. Wiley Online Library. 10.1002/anie.199526131

[B16] Corey E. J. Wipke W. T. (1969). Computer-assisted design of complex organic syntheses. Science 166 (3902), 178–192. 10.1126/science.166.3902.178 17731475

[B17] Corey E. J. (1967). General methods for the construction of complex molecules. Pure Appl. Chem. 14 (1), 19–38. 10.1351/pac196714010019.1

[B18] Coulom R. (2006). “Efficient selectivity and backup operators in Monte-Carlo tree search,” in Computers and games. 4630, Van Den Herik H. J. Ciancarini P. Donkers H. H. L. M. Berlin, Heidelberg: Springer Berlin Heidelberg. 72–83. 10.1007/978-3-540-75538-8_7

[B19] Deming S. N. Pardue H. L. (1971). Automated instrumental system for fundamental characterization of chemical reactions. Anal. Chem. 43 (2), 192–200. 10.1021/ac60297a001

[B20] Destro F. Inguva P. K. Srisuma P. Braatz R. D. (2024). Advanced methodologies for model-based optimization and control of pharmaceutical processes. Curr. Opin. Chem. Eng. 45, 101035. 10.1016/j.coche.2024.101035

[B21] Dhakal A. McKay C. Tanner J. J. Cheng J. (2022). Artificial intelligence in the prediction of protein–ligand interactions: recent advances and future directions. Briefings Bioinforma. 23 (1), bbab476. 10.1093/bib/bbab476 34849575 PMC8690157

[B22] DiMasi J. A. Grabowski H. G. Hansen R. W. (2016). Innovation in the pharmaceutical industry: new estimates of R&D costs. J. Health Econ. 47, 20–33. 10.1016/j.jhealeco.2016.01.012 26928437

[B23] Elman J. L. (1990). Finding structure in time. Cognitive Sci. 14 (2), 179–211. 10.1016/0364-0213(90)90002-E

[B24] European Medicines Agency (EMA) (2024). Reflection paper on the use of artificial intelligence (AI) in the medicinal product lifecycle. EMA Reflect. Pap. Ref. EMA/CHMP/358838/2021. Available online at: https://www.ema.europa.eu/en/documents/scientific-guideline/reflection-paper-use-artificial-intelligence-ai-medicinal-product-lifecycle_en.pdf

[B25] Ferreira L. L. G. Andricopulo A. D. (2019). ADMET modeling approaches in drug discovery. Drug Discov. Today 24 (5), 1157–1165. 10.1016/j.drudis.2019.03.015 30890362

[B26] Ferruz N. Schmidt S. Höcker B. (2022). ProtGPT2 is a deep unsupervised language model for protein design. Nat. Commun. 13 (1), 4348. 10.1038/s41467-022-32007-7 35896542 PMC9329459

[B27] Folgado F. J. Calderón D. González I. Calderón A. J. (2024). Review of industry 4.0 from the perspective of automation and supervision systems: definitions, architectures and recent trends. Electronics 13 (4). 10.3390/electronics13040782

[B28] Gnoth S. Jenzsch M. Simutis R. Lübbert A. (2007). Process analytical Technology (PAT): Batch-to-batch reproducibility of fermentation processes by robust process operational design and control. J. Biotechnol. 132 (2), 180–186. 10.1016/j.jbiotec.2007.03.020 17559961

[B29] Gordon D. E. Jang G. M. Bouhaddou M. Xu J. Obernier K. White K. M. (2020). A SARS-CoV-2 protein interaction map reveals targets for drug repurposing. Nature 583 (7816), 459–468. 10.1038/s41586-020-2286-9 32353859 PMC7431030

[B2] Gorle A. Pawar S. Dipali A. Nimbarthe H. Leena C. (2025). AI and Machine Learning in Pharmaceutical Manufacturing: Revolutionizing Process Optimization. J. Drug Delivery 15 (10).

[B30] Granda J. M. Donina L. Dragone V. Long D.-L. Cronin L. (2018). Controlling an organic synthesis robot with machine learning to search for new reactivity. Nature 559 (7714), 377–381. 10.1038/s41586-018-0307-8 30022133 PMC6223543

[B31] Green chemistry. (2024). Green chemistry articles of interest to the pharmaceutical industry. Organic Process Research & Development, vol. 28, no. 9, 3450–3459. 10.1021/acs.oprd.4c00300

[B32] Gu M. Sun S. You Q. Wang L. (2023). Forward or backward: lessons learned from small molecule drugs approved by FDA from 2012 to 2022. Molecules 28 (24), 7941. 10.3390/molecules28247941 38138431 PMC10745639

[B33] Guo J. Yu C. Li K. Zhang Y. Wang G. Li S. (2024). Retrosynthesis zero: self-improving global synthesis planning using reinforcement learning. J. Chem. Theory Comput. 20 (11), 4921–4938. 10.1021/acs.jctc.4c00071 38747149

[B34] Haigney S. (2022). Controlling quality in aging facilities. Available online at: https://www.biopharminternational.com/view/controlling-quality-in-aging-facilities (Accessed: June 08, 2025).

[B35] Han P. Zhao P. Lu C. Huang J. Wu J. Shang S. (2022a). GNN-Retro: retrosynthetic planning with graph neural networks. Proc. AAAI Conf. Artif. Intell. 36 (4), 1–9. 10.1609/aaai.v36i4.20318

[B36] Han H. Zhao P. Lu C. Huang J. Wu J. Shang S. (2022b). “Gnn-retro: retrosynthetic planning with graph neural networks.”Proceedings of the AAAI conference on artificial Intelligence. 36. Palo Alto: AAAI Press, 4014–4021. 10.1609/aaai.v36i4.20455

[B37] Harwood L. A. Xiong Z. Christensen K. E. Wang R. Wong L. L. Robertson J. (2023). Selective P450BM3 hydroxylation of cyclobutylamine and Bicyclo [1.1.1]pentylamine derivatives: underpinning synthetic chemistry for drug discovery. J. Am. Chem. Soc. 145 (50), 27767–27773. 10.1021/jacs.3c10542 38051939 PMC10740007

[B38] Hyer A. Gregory D. Kay K. Le Q. Turnage J. Gupton F. (2024). Continuous manufacturing of active pharmaceutical ingredients: current trends and perspectives. Adv. Synthesis & Catal. 366 (3), 357–389. 10.1002/adsc.202301137

[B39] ICH Q8. ICH Q8 (R2) pharmaceutical development - scientific guideline. (2024). European Medicines Agency EMA. Accessed: August. 27, 2024. Available online at: https://www.ema.europa.eu/en/ich-q8-r2-pharmaceutical-development-scientific-guideline

[B40] International Conference on Harmonisation (2014). International conference on harmonisation (ICH), “Q8 (R2) Pharmaceutical development - scientific guideline,” ICH guideline, Ref. EMA/CHMP/ICH/167068/2004. Available online at: https://www.ich.org/page/quality-guidelines.

[B41] Jia C.-Y. Li J.-Y. Hao G.-F. Yang G.-F. (2020). A drug-likeness toolbox facilitates ADMET study in drug discovery. Drug Discov. Today 25 (1), 248–258. 10.1016/j.drudis.2019.10.014 31705979

[B42] Jiang W. Zhao Z. (2025). Trends in research on AI-aided drug discovery from 2009 to 2023: a 15-year bibliometric analysis. *Intelligent Pharmacy* 3 (1), 71–83. 10.1016/j.ipha.2024.09.001

[B43] Jiang Z. Cao J. (2022). Analysis of optimization countermeasures of pharmaceutical and chemical production quality management. J. Project Manage 3 (10), 166–168. 10.12238/jpm.v3i10.5370

[B44] Jiang S. (2012). Computer-aided synthesis Planning: a brief history.

[B45] Kim D.-G. Choi J.-Y. (2021). Optimization of design parameters in LSTM model for predictive maintenance. Applied Sciences 11 (14), 6450. 10.3390/app11146450

[B46] Lab-scale experiment (2025). Lab-scale experiment vs. pilot-scale experiment - what’s the difference?” Accessed: June. 16, 2025. Available online at: https://thisvsthat.io/lab-scale-experiment-vs-pilot-scale-experiment

[B47] Lalmuanawma S. Hussain J. Chhakchhuak L. (2020). Applications of machine learning and artificial intelligence for Covid-19 (SARS-CoV-2) pandemic: a review. Chaos, Solitons & Fractals 139, 110059. 10.1016/j.chaos.2020.110059 32834612 PMC7315944

[B48] LeCun Y. Boser B. Denker J. Henderson D. Howard R. Hubbard W. (1989). “Handwritten digit recognition with a back-propagation network,” in Advances in neural information processing systems (Paris, France: Morgan-Kaufmann). Available online at: https://proceedings.neurips.cc/paper_files/paper/1989/hash/53c3bce66e43be4f209556518c2fcb54-Abstract.html (Accessed: April 08, 2025).

[B49] Lee S. L. O’Connor T. F. Yang X. Cruz C. N. Chatterjee S. Madurawe R. D. (2015). Modernizing pharmaceutical manufacturing: from batch to continuous production. J Pharm Innov 10 (3), 191–199. 10.1007/s12247-015-9215-8

[B50] Lee S.-H. Kim J.-K. Jee J.-P. Jang D.-J. Park Y.-J. Kim J.-E. (2022). Quality by design (QbD) application for the pharmaceutical development process. J. Pharm. Investig. 52 (6), 649–682. 10.1007/s40005-022-00575-x

[B51] Li H. Chiang A. W. T. Lewis N. E. (2022). Artificial intelligence in the analysis of glycosylation data. Biotechnology Advances 60, 108008. 10.1016/j.biotechadv.2022.108008 35738510 PMC11157671

[B52] Lin K. Xu Y. Pei J. Lai L. (2020). Automatic retrosynthetic route planning using template-free models. Chem. Sci. 11 (12), 3355–3364. 10.1039/C9SC03666K 34122843 PMC8152431

[B53] Luo J. Wang Y. Su Q. Yu Q. Zhai X. Zou Y. (2024). Rapid and sustainable production of nano and micro medicine crystals via freeze-dissolving technology. Powder Technology 443, 119913. 10.1016/j.powtec.2024.119913

[B54] Maddikunta P. K. R. Pham Q. V. Deepa N. Dev K. Gadekallu T. R. Ruby R. (2022). Industry 5.0: a survey on enabling technologies and potential applications. Journal of Industrial Information Integration 26, 100257. 10.1016/j.jii.2021.100257

[B55] Markey C. Croset S. Woolley O. R. Buldun C. M. Koch C. Koller D. (2024). Characterizing emerging companies in computational drug development. Nat Comput. Sci 4 (2), 96–103. 10.1038/s43588-024-00594-8 38413778

[B56] Mitra A. (2024). Cellular automata-based MapReduce design: migrating a big data processing model from industry 4.0 to industry 5.0. E-Prime—Advances in Electrical Engineering, Electronics and Energy 8, 100603. 10.1016/j.prime.2024.100603

[B57] Montgomery D. C. (2019). Introduction to statistical quality control. 8th ed. Hoboken, NJ, USA: John Wiley & Sons.

[B58] Moore M. (2019). What is industry 4.0? Everything you need to know. Ang. A TechRadar Available online at: https://www.techradar.com/news/what-is-industry-40-everything-you-need-to-know.

[B59] Muhaimin M. Chaerunisaa A. Y. Bodmeier R. (2021). Real-time particle size analysis using focused beam reflectance measurement as a process analytical technology tool for continuous microencapsulation process. Sci. Rep. 11 (1), 19390. 10.1038/s41598-021-98835-9 34588571 PMC8481503

[B60] Munir N. Nugent M. Whitaker D. McAfee M. (2021). Machine learning for process monitoring and control of hot-melt extrusion: current state of the art and future directions. Pharmaceutics 13 (9), 1432. 10.3390/pharmaceutics13091432 34575508 PMC8466632

[B61] Nagy B. Petra D. Galata D. L. Démuth B. Borbás E. Marosi G. (2019). Application of artificial neural networks for process analytical Technology-based dissolution testing. International Journal of Pharmaceutics 567, 118464. 10.1016/j.ijpharm.2019.118464 31252145

[B62] Neugebauer P. Zettl M. Moser D. Poms J. Kuchler L. Sacher S. (2024). Process analytical technology in downstream-processing of Drug Substances– A review. International Journal of Pharmaceutics 661, 124412. 10.1016/j.ijpharm.2024.124412 38960339

[B63] Orehek J. Teslić D. Likozar B. (2021). Continuous crystallization processes in pharmaceutical manufacturing: a review. Org. Process Res. Dev. 25 (1), 16–42. 10.1021/acs.oprd.0c00398

[B64] Paul S. M. Mytelka D. S. Dunwiddie C. T. Persinger C. C. Munos B. H. Lindborg S. R. (2010). How to improve R&D productivity: the pharmaceutical industry’s grand challenge. Nat Rev Drug Discovery 9 (3), 203–214. 10.1038/nrd3078 20168317

[B65] Pflüger P. M. Glorius F. (2020). Molecular machine learning: the future of synthetic chemistry? Angewandte Chemie International Edition 59 (43), 18860–18865. 10.1002/anie.202008366 32931084

[B66] PotentialNet for Molecular Property Prediction (2025). PotentialNet for molecular property prediction. ACS Central Science. 10.1021/acscentsci.8b00507 PMC6276035 30555904

[B67] Prueksaritanont T. Tang C. (2012). ADME of Biologics—what have we learned from small molecules? AAPS J 14 (3), 410–419. 10.1208/s12248-012-9353-6 22484625 PMC3385832

[B68] Rada M. (2015). INDUSTRY 5.0 - from virtual to physical. LinkedIn. Available online at: https://www.linkedin.com/pulse/industry-50-from-virtual-physical-michael-rada.

[B69] Rada M. (2020). Industry 5.0 definition. Medium. Available online at: https://michael-rada.medium.com/industry-5-0-definition-6a2f9922dc48.

[B70] Raies A. B. Bajic V. B. (2016). *In silico* toxicology: computational methods for the prediction of chemical toxicity. Wiley Interdiscip. Rev. Comput. Mol. Sci. 6, 147–172. 10.1002/wcms.1240 27066112 PMC4785608

[B71] Rayyad S. E. Massot V. Chourpa I. (2023). Comparison of SVMR and PLSR for ATR-IR data treatment: application to AQC of mAbs in clinical solutions. Vib. Spectrosc. 129, 103594. 10.1016/j.vibspec.2023.103594

[B72] ReactionMap: An Efficient Atom (2025). ReactionMap: an efficient atom-mapping algorithm for chemical reactions. Journal of Chemical Information and Modeling. 10.1021/ci400326p 24160861

[B73] Rich S. Venkatasubramanian V. (1987). Model-based reasoning in diagnostic expert systems for chemical process plants. Comput. Chem. Eng. 11 (2), 111–122. 10.1016/0098-1354(87)80012-1

[B74] Roggo Y. Chalus P. Maurer L. Lema-Martinez C. Edmond A. Jent N. (2007). A review of near infrared spectroscopy and chemometrics in pharmaceutical technologies. J. Pharm. Biomed. Anal. 44, 683–700. 10.1016/j.jpba.2007.03.030 17482417

[B75] Sadybekov A. V. Katritch V. (2023). Computational approaches streamlining drug discovery. Nature 616 (7958), 673–685. 10.1038/s41586-023-05905-z 37100941

[B76] Sadybekov A. A. Sadybekov A. V. Liu Y. Iliopoulos-Tsoutsouvas C. Huang X. P. Pickett J. (2022). Synthon-based ligand discovery in virtual libraries of over 11 billion compounds. Nature 601 (7893), 452–459. 10.1038/s41586-021-04220-9 34912117 PMC9763054

[B77] Sarkis M. Bernardi A. Shah N. Papathanasiou M. M. (2021). Emerging challenges and opportunities in pharmaceutical manufacturing and distribution. Processes 9 (3), 457. 10.3390/pr9030457

[B78] Schwaller P. Laino T. Gaudin T. Bolgar P. Hunter C. A. Bekas C. (2019). Molecular transformer: a model for uncertainty-calibrated chemical reaction prediction. ACS Cent. Sci. 5 (9), 1572–1583. 10.1021/acscentsci.9b00576 31572784 PMC6764164

[B79] Segler M. H. S. Waller M. P. (2017). Neural-symbolic machine learning for retrosynthesis and reaction prediction. Chem. Eur. J. 23 (25), 5966–5971. 10.1002/chem.201605499 28134452

[B80] Segler M. H. S. Preuss M. Waller M. P. (2018). Planning chemical syntheses with deep neural networks and symbolic AI. Nature 555 (7698), 604–610. 10.1038/nature25978 29595767

[B81] Shandong Institute of Industrial Technology for Health Sciences and Medicine (2025). Accessed: June. 16, 2025. Available online at: https://en.siit.org.cn/index.php?m=home&c=Lists&a=index&tid=121

[B82] Sharma A. Kaur J. Singh I. (2020). Internet of things (IoT) in pharmaceutical manufacturing, warehousing, and supply chain management. SN Comput. SCI. 1 (4), 232. 10.1007/s42979-020-00248-2

[B83] Sharma D. Patel P. Shah M. (2023). A comprehensive study on industry 4.0 in the pharmaceutical industry for sustainable development. Environ Sci Pollut Res 30 (39), 90088–90098. 10.1007/s11356-023-26856-y 37129827 PMC10153053

[B84] Sharma R. Jesubalan N. G. Rathore A. S. (2024). Application of ensemble learning to augment fluorescence-based PAT and enable real-time monitoring of protein refolding. Biochemical Engineering Journal 204, 109252. 10.1016/j.bej.2024.109252

[B85] Singh R. Sahay A. Karry K. M. Muzzio F. Ierapetritou M. Ramachandran R. (2014). Implementation of an advanced hybrid MPC–PID control system using PAT tools into a direct compaction continuous pharmaceutical tablet manufacturing pilot plant. International Journal of Pharmaceutics 473 (1), 38–54. 10.1016/j.ijpharm.2014.06.045 24974987

[B86] Southey M. W. Y. Brunavs M. (2023). Introduction to small molecule drug discovery and preclinical development. Front. Drug Discov. 3 (Nov), 1314077. 10.3389/fddsv.2023.1314077

[B87] Steiner S. Wolf J. Glatzel S. Andreou A. Granda J. M. Keenan G. (2019). Organic synthesis in a modular robotic system driven by a chemical programming language. Science 363 (6423), eaav2211. 10.1126/science.aav2211 30498165

[B88] Sun Y. Wang C. Yang P. Yue J. Y. Xu C. Zhou J. S. (2023). Hydrogen bond enhanced enantioselectivity in the nickel-catalyzed transfer hydrogenation of α-Substituted acrylic acid with formic acid. ACS Catal. 13 (21), 14213–14220. 10.1021/acscatal.3c04187

[B89] Supercritical fluid (2021). Supercritical fluid (SCF)-assisted fabrication of carrier-free drugs: an eco-friendly welcome to active pharmaceutical ingredients (APIs). Advanced Drug Delivery Reviews. 176, p. 113846. 10.1016/j.addr.2021.113846 34197896

[B90] Suriyaamporn P. Pamornpathomkul B. Patrojanasophon P. Ngawhirunpat T. Rojanarata T. Opanasopit P. (2024). The artificial intelligence-powered new era in pharmaceutical research and development: a review. AAPS PharmSciTech 25 (6), 188. 10.1208/s12249-024-02901-y 39147952

[B91] Tian S. Wang J. Li Y. Li D. Xu L. Hou T. (2015). The application of *in silico* drug-likeness predictions in pharmaceutical research. Advanced Drug Delivery Reviews 86, 2–10. 10.1016/j.addr.2015.01.009 25666163

[B92] Tianfu International Biocity (2025). Focusing on high-end chemical preparations, this pilot-scale platform has achieved a big internationalization. Accessed: June. 16, 2025. Available online at: https://www.cdht.gov.cn/cdht/c140946/2023-04/07/content_9f474fdf4efe45c28cf9e14022d58ef2.shtml

[B93] Troup G. M. Georgakis C. (2013). Process systems engineering tools in the pharmaceutical industry. Computers & Chemical Engineering 51, 157–171. 10.1016/j.compchemeng.2012.06.014

[B94] U.S. Food and Drug Administration (2004). PAT — a framework for innovative pharmaceutical development, manufacturing, and quality assurance. Guidance for Industry. Available online at: http://www.fda.gov/cder/guidance/index.htm.

[B95] U.S. Food and Drug Administration (FDA) (2021). Advancing emerging technology applications for pharmaceutical innovation and modernization. Rockville, MD: FDA Guidance for Industry. Available online at: https://www.fda.gov/media/146810/download.

[B96] Uraikul V. Chan C. W. Tontiwachwuthikul P. (2007). Artificial intelligence for monitoring and supervisory control of process systems. Engineering Applications of Artificial Intelligence 20 (2), 115–131. 10.1016/j.engappai.2006.07.002

[B97] van Erp T. Carvalho N. G. P. Gerolamo M. C. Gonçalves R. Rytter N. G. M. Gladysz B. (2024). Industry 5.0: a new strategy framework for sustainability management and beyond. Journal of Cleaner Production 461, 142271. 10.1016/j.jclepro.2024.142271

[B98] Vasconcellos E. P. Souza P. M. T. G. D. Franco M. R. Castro V. G. D. Souza L. V. Lago R. M. (2021). Escalonamento de tecnologias: desenvolvimento de produto e processo do laboratório à escala piloto conectado ao mercado (parte 1). Quím. Nova 44, 377–384. 10.21577/0100-4042.20170665

[B99] Vaswani A. Shazeer N. Parmar N. Uszkoreit J. Jones L. Gomez A. N. (2017). Attention is all you need in Presented at the neural information processing systems. Available online at: https://www.semanticscholar.org/paper/Attention-is-All-you-Need-Vaswani-Shazeer/204e3073870fae3d05bcbc2f6a8e263d9b72e776 (Accessed: August 16, 2024).

[B100] Vega-Zambrano C. Diangelakis N. A. Charitopoulos V. M. (2025). Data-driven model predictive control for continuous pharmaceutical manufacturing. Int. J. Pharm. 672, 125322. 10.1016/j.ijpharm.2025.125322 39921017

[B101] Wang Z. Zhao W. Hao G.-F. Song B.-A. (2020). Automated synthesis: current platforms and further needs. Drug Discovery Today 25 (11), 2006–2011. 10.1016/j.drudis.2020.09.009 32949527

[B102] Wang W. Ye Z. Gao H. Ouyang D. (2021a). Computational pharmaceutics - a new paradigm of drug delivery. Journal of Controlled Release 338, 119–136. 10.1016/j.jconrel.2021.08.030 34418520

[B103] Wang Z. Zhao W. Hao G. Song B. (2021b). Mapping the resources and approaches facilitating computer-aided synthesis planning. Org. Chem. Front. 8 (4), 812–824. 10.1039/D0QO00946F

[B104] Wang Z. Zhang W. Liu B. (2021c). Computational analysis of synthetic planning: past and future. Chinese Journal of Chemistry 39 (11), 3127–3143. 10.1002/cjoc.202100273

[B105] Wang G. Ang H. T. Dubbaka S. R. O’Neill P. Wu J. (2023a). Multistep automated synthesis of pharmaceuticals. Trends in Chemistry 5 (6), 432–445. 10.1016/j.trechm.2023.03.008

[B106] Wang G. Wu X. Xin B. Gu X. Wang G. Zhang Y. (2023b). Machine learning in unmanned systems for chemical synthesis. Molecules 28 (5), 2232. 10.3390/molecules28052232 36903478 PMC10004533

[B107] Wong C. H. Siah K. W. Lo A. W. (2019). Estimation of clinical trial success rates and related parameters. Biostatistics 20 (2), 273–286. 10.1093/biostatistics/kxx069 29394327 PMC6409418

[B108] Yang H. Hu Y. Zou Y. Zhang Z. Zhang W. (2023). Cobalt-catalyzed efficient asymmetric hydrogenation of α-Primary amino ketones. JACS Au 3 (11), 2981–2986. 10.1021/jacsau.3c00524 38034968 PMC10685343

[B109] You M.-Y. Meng G. (2012). A modularized framework for predictive maintenance scheduling. Proceedings of the Institution of Mechanical Engineers, Part O Journal of Risk and Reliability 226 (4), 380–391. 10.1177/1748006X11431209 21953325

[B110] Záhonyi P. Fekete D. Szabó E. Nagy Z. K. Nagy B. (2025). Explainable artificial neural network as a soft sensor to predict the moisture content in a continuous granulation line. Eur. J. Pharm. Sci. 212, 107173. 10.1016/j.ejps.2025.107173 40550371

[B111] Zeng X. Xiang H. Yu L. Wang J. Li K. Nussinov R. (2022). Accurate prediction of molecular properties and drug targets using a self-supervised image representation learning framework. Nat Mach Intell 4 (11), 1004–1016. 10.1038/s42256-022-00557-6

[B112] Zhang L. Mao S. (2017). Application of quality by design in the current drug development. Asian Journal of Pharmaceutical Sciences 12 (1), 1–8. 10.1016/j.ajps.2016.07.006 32104308 PMC7032183

[B113] Zhang M. (2023). “Pilot-scale studies, scaling-up, and technology transfer,” in Iron ores bioprocessing. Editor Zhang M. (Cham: Springer International Publishing), 161–167. 10.1007/978-3-031-10102-1_9

[B114] Zhao S. Li C. Hou X. (2023). An analysis of the issues and countermeasures in the construction of pilot testing platforms for the transformation of scientific and technological achievements. Enterprise Reform and Management (20), 45–47. 10.13768/j.cnki.cn11-3793/f.2023.1127

[B115] Zhu Z. Lin K. Jain A. K. Zhou J. (2023). Transfer learning in deep Reinforcement learning: a survey. IEEE Transactions on Pattern Analysis and Machine Intelligence 45 (11), 13344–13362. 10.1109/TPAMI.2023.3292075 37402188 PMC11018366

